# Integrating Ligand and Target-Driven Based Virtual Screening Approaches With *in vitro* Human Cell Line Models and Time-Resolved Fluorescence Resonance Energy Transfer Assay to Identify Novel Hit Compounds Against BCL-2

**DOI:** 10.3389/fchem.2020.00167

**Published:** 2020-04-09

**Authors:** Gurbet Tutumlu, Berna Dogan, Timucin Avsar, Muge Didem Orhan, Seyma Calis, Serdar Durdagi

**Affiliations:** ^1^Computational Biology and Molecular Simulations Laboratory, Department of Biophysics, School of Medicine, Bahcesehir University, Istanbul, Turkey; ^2^Department of Medical Biology, Bahcesehir University, School of Medicine, Istanbul, Turkey; ^3^Neuroscience Program, Health Sciences Institute, Bahcesehir University, Istanbul, Turkey; ^4^Neuroscience Laboratory, Health Sciences Institute, Bahcesehir University, Istanbul, Turkey; ^5^Molecular Biology, Genetics and Biotechnology Graduate Program, Istanbul Technical University, Istanbul, Turkey

**Keywords:** BCL-2, molecular docking, MD simulations, virtual screening, text mining, *in vitro* human cell line models, TR-FRET assay, binary QSAR models

## Abstract

Antiapoptotic members of *B*-*cell leukemia*/*lymphoma*-2 (BCL-2) family proteins are one of the overexpressed proteins in cancer cells that are oncogenic targets. As such, targeting of BCL-2 family proteins raises hopes for new therapeutic discoveries. Thus, we used multistep screening and filtering approaches that combine structure and ligand-based drug design to identify new, effective BCL-2 inhibitors from a small molecule database (Specs SC), which includes more than 210,000 compounds. This database is first filtered based on binary “*cancer-QSAR”* model constructed with 886 training and 167 test set compounds and common 26 toxicity quantitative structure-activity relationships (QSAR) models. Predicted non-toxic compounds are considered for target-driven studies. Here, we applied two different approaches to filter and select hit compounds for further *in vitro* biological assays and human cell line experiments. In the first approach, a molecular docking and filtering approach is used to rank compounds based on their docking scores and only a few top-ranked molecules are selected for further long (100-ns) molecular dynamics (MD) simulations and *in vitro* tests. While docking algorithms are promising in predicting binding poses, they can be less prone to precisely predict ranking of compounds leading to decrease in the success rate of *in silico* studies. Hence, in the second approach, top-docking poses of each compound filtered through QSAR studies are subjected to initially short (1 ns) MD simulations and their binding energies are calculated via molecular mechanics generalized Born surface area (MM/GBSA) method. Then, the compounds are ranked based on their average MM/GBSA energy values to select hit molecules for further long MD simulations and *in vitro* studies. Additionally, we have applied text-mining approaches to identify molecules that contain “*indol*” phrase as many of the approved drugs contain indole and indol derivatives. Around 2700 compounds are filtered based on “*cancer-QSAR”* model and are then docked into BCL-2. Short MD simulations are performed for the top-docking poses for each compound in complex with BCL-2. The complexes are again ranked based on their MM/GBSA values to select hit molecules for further long MD simulations and *in vitro* studies. In total, seven molecules are subjected to biological activity tests in various human cancer cell lines as well as Time-Resolved Fluorescence Resonance Energy Transfer (TR-FRET) assay. Inhibitory concentrations are evaluated, and biological activities and apoptotic potentials are assessed by cell culture studies. Four molecules are found to be limiting the proliferation capacity of cancer cells while increasing the apoptotic cell fractions.

## Introduction

Finding a cure for cancer is still a challenging task, despite the understanding of molecular mechanisms and causal relationships participating in the pathology of cancer since the mid-1980s (Fesik, 2005). As stated by Hanahan and Weinberg, multistage development of tumors consists of six biological features widely known as hallmarks of cancer: (i) maintaining proliferative signaling, (ii) avoiding growth suppressors, (iii) triggering invasion and metastasis, (iv) empowering replicative perpetuity, (v) inducing angiogenesis, and (vi) resisting cell death (Hanahan and Weinberg, 2000, 2011). The ability of cancer cells to escape from programmed cell death, namely, apoptosis, remains a critical feature of these six indicators (Mohamad Rosdi et al., 2018). Apoptosis is a molecular pathway that results with self-destruction of the cell, either following termination of physiological function or after a crucial damage to genetic material (Igney and Krammer, 2002; Reed, 2002; Verma et al., 2015). The well-defined basic apoptosis pathways, extrinsic and the intrinsic pathways, are variously stimulated, and they use determined signaling elements (Kollek et al., 2016). The extrinsic pathway is activated by outer stimulation of death receptors. Death receptors are members of the tumor necrosis factor (TNF) receptor family, which has an intracellular death domain that is able to accumulate and trigger caspase-8 followed by operation of effector caspases including caspase-3, -6, or -7 (Youle and Strasser, 2008; Eimon and Ashkenazi, 2010; Wu et al., 2018). The intrinsic pathway, also called mitochondrial pathway, is initiated by a variety of cytotoxic damages or growth signals, some of which are genetic instability, inadequate developmental stimulation, and invasion by viral pathogens. *B*-*cell leukemia*/*lymphoma*-2 (BCL-2) family proteins tightly regulate this process and subsequently leads to the activation of caspase-9 (Cory et al., 2003; Youle and Strasser, 2008; Eimon and Ashkenazi, 2010).

All members of BCL-2 protein family have retained sequence patterns regarded as the BCL-2 homology (BH) domains and could be divided into three main classes. The first class of proteins are made up of the proapoptotic activator BH domain 3 (BH3) only proteins such as BIM, BID, and PUMA. Immediately upon their activation, they serve as molecular guardians that connect outer spurs to the mitochondrial pathway. The following group contains the proapoptotic effectors, which are multidomain proteins, such as BAX and BAK, and each of them has three BH domains. These proteins distort the integrity of mitochondrial outer membrane, which leads to free movement of cytochrome C to cytoplasm, initiates downstream caspase activity, and ultimately to trigger the termination of cells. The last class of BCL-2 family are the antiapoptotic protein, BCL-XL, BCL-2, MCL-1, etc. All of these members consist of four BH domains and keeps cells safe by segregating their proapoptotic peers. The most important point in promoting apoptosis is to increase the amount of BH3-only proteins or switch off one of its antiapoptotic BCL-2 counterparts (Fesik, 2005; Dewson and Kluck, 2010; Chung, 2018; Mohamad Rosdi et al., 2018). The idea of BH3 mimetics as promising anticancer drugs is inspired by the conclusion that a great deal of cancers rely on BCL-2 family proteins and that the interaction between these proteins occurs through specific BH domains (Oltersdorf et al., 2005; Soderquist and Eastman, 2016). A genuine BH3 mimetic is expected to imitate the BH3 domain of a proapoptotic BCL-2 protein, thus deactivating the antiapoptotic family members by filling up their BH3-binding pockets.

Apoptotic cell death is an innate hurdle to growth of tumor cells; hence, one of the fundamental hallmarks of cancer cells is the avoidance of apoptosis, which comprises a crucial process in resistance to chemotherapeutics. This phenomenon led to peculiar approaches in anticancer therapies focusing on apoptosis such as suppression of survival factors that are detected to be overexpressed in numerous malignancies. In the group of survival factors, BCL-2 proteins are one of the families that step forward for drug discovery studies (Lessene et al., 2008; Hanahan and Weinberg, 2011; Billard, 2013). For example, a small molecule, named ABT-737, was issued as a potential inhibitor of BCL-2 and BCL-XL, which occupies their BH3-binding domain and further triggers apoptosis in diversified cancer types (Tse et al., 2008; Soderquist and Eastman, 2016). Ensuing pharmaceutical trials guided to clinical studies with ABT-263 (navitoclax), which had boosted bioavailability and indicated efficacy in leukemia and a few other neoplasias. However, they also manifested toxicities such as neutropenia and thrombocytopenia, leading to dose limitations (Tse et al., 2008; Gandhi et al., 2011). The thrombocytopenia was connected to the blockage of BCL-XL, as BCL-XL is essential for survival of platelets (Zhang et al., 2007). More recently, ABT-199 (venetoclax) was designed as a selective BCL-2 inhibitor and it evades the issue of thrombocytopenia (Souers et al., 2013). However, it also carries some side effects such as diarrhea, nausea, low white blood cell counts, high K^+^ ion concentrations in the blood, headache, etc. Thus, novel BCL-2 inhibitors with better pharmacodynamic as well as pharmacokinetic profiles are needed.

It is well-established, especially in the last years, that taking a new drug into the market is both a time consuming and costly process. As a result, computer-aided drug design techniques have become prominent in drug development process (Lionta et al., 2014; Yoshino et al., 2015, 2017; Chiba et al., 2017; Halim et al., 2017; Durdagi et al., 2018a, 2019; Fu et al., 2018; Is et al., 2018; Mirza et al., 2018; Erol et al., 2019; Zaka et al., 2019). Different strategies depending on the availability of target molecules have been developed; structure-based drug design in which the target structure is known and ligand-based drug design that could be applied in cases that target structure is not known. It is also possible to combine both approaches to increase the possibility of “hit molecule” discovery as has been done in our previous studies (Durdagi et al., 2018a,b; Zaka et al., 2018; Kanan et al., 2019; Mollica et al., 2019). The most widely used technique in target-driven-based drug design is the molecular docking, and there are various docking programs as well as many different scoring functions to rank binding poses. Large molecule libraries can be screened using high throughput virtual screening, and lead compounds can be identified for further studies, quickly. By the use of more sophisticated docking algorithms and scoring functions, binding modes of compounds to target can also be determined. However, as expected, each of the docking algorithms and scoring functions have their own strengths and weaknesses. Numerous studies have been conducted to evaluate the comparative assessment of the docking and scoring functions (Bissantz et al., 2000; Bursulaya et al., 2003; Chen et al., 2006; Warren et al., 2006; Cross et al., 2009; Li et al., 2014). The latest evaluation study was conducted by Li et al. for 20 scoring functions on a diverse set of protein–ligand complexes (Li et al., 2014). Their comparison of scoring functions was based on four aspects: “scoring power” (binding affinity prediction), “ranking power” (relative ranking prediction), “docking power” (binding pose prediction), and “screening power” (discrimination of true binders from random molecules). Their results showed that scoring functions were generally more promising in docking and screening power tests than scoring and ranking power tests. In addition, scoring functions that were among top-ranked in docking power test were also more successful in screening power test but poor in other two power tests. This study, which shows that every scoring function has its own weaknesses, has represented that the ordering of compounds only by their docking scores may not accomplish the correct ranking of compounds; hence, if the molecules will only be selected according to their top docking scores for further studies such as *in vitro* tests, this may lead to false positive results (Rastelli et al., 2009; Rastelli and Pinzi, 2019). Therefore, in this study, we use another approach in ranking compounds that is based on molecular dynamics (MD) simulations and molecular mechanics generalized Born surface area (MM/GBSA) calculations after initial pose prediction by molecular docking.

In the present study, in order to identify novel BCL-2 inhibitors, ligand- and target-driven-based techniques were integrated with text mining approach, and novel hit molecules were identified with the virtual screening of small molecules library (Specs SC) that includes more than 212,000 compounds. In the identification of hits, two different approaches were considered: (i) Compounds were ranked by their docking scores, and MD simulations for 100 ns were carried out for the selected compounds and average MM/GBSA energies were calculated; (ii) Short (1-ns) MD simulations were applied for top-docking poses of all selected 342 compounds from binary quantitative structure-activity relationships (QSAR) models, and average MM/GBSA scores from short MD simulations were calculated. The average MM/GBSA scores were considered in the selection of compounds for longer MD simulations (100 ns) followed by MM/GBSA calculations. Additionally, it is known that many currently used Food and Drug Administration (FDA)-approved chemotherapeutics include indole fragment. To increase the probability of discovering hit molecules with potential anticancer properties, we screened Specs-SC database to identify molecules that contain “*indol”* groups by using text mining. Around 2700 compounds were screened against BCL-2, and novel hits that includes “*indol”* fingerprints were identified.

## Materials and Methods

### Binary QSAR Models

MetaCore/MetaDrug (MC/MD) platform from Clarivate Analytics provides a comprehensive tool to analyze the pharmacodynamic and pharmacokinetic profiles for screening molecules. Using MC/MD, it is possible to calculate “therapeutic activity values (TAV)” of molecules for 25 common diseases including cancer by binary QSAR disease models. Additionally, toxicities of compounds could also be predicted in 26 different toxicity QSAR models using MC/MD. The *Tanimoto Prioritization (TP)* feature was applied to detect similarity between compounds and training and test set molecules analyzed in QSAR models based on fragments within the structure. QSAR models in the platform were constructed using various compounds based on experimental evidence of their activity/function on a particular protein of interest and then tested with validation sets. Estimated QSAR values (normalized between 0 and 1) >0.5 indicate potential therapeutic activity. The details about QSAR models could be found in the following reference (Kanan et al., 2019). In the current study, we used “*cancer-QSAR”* model, which has the following parameters: Training set *N* = 886, Test set *N* = 167, Sensitivity = 0.89, Specificity = 0.83, Accuracy = 0.86, MCC = 0.72.

### Ligand Preparation and Protein Preparation

The compounds screened in this study (212, 520 molecules) were downloaded from Specs SC database (https://www.specs.net/index.php). 2D structures were used in binary QSAR models both for therapeutic activity prediction and toxicity prediction. After the target-driven screening and toxicity tests, 250 compounds were filtered and these molecules were prepared with the OPLS2005 forcefield using LigPrep module (Schrödinger Release 2015-2, 2015) of Maestro program (Banks et al., 2005). The possible ionization states at neutral pH 7.4 was determined by Epik module (Shelley et al., 2007). All possible tautomers as well as stereoisomers (if any) were generated. At the end, 342 structures were obtained and used in further docking and MD simulations. Two structures of BCl-2 solved by X-ray diffraction [Protein Data Bank (PDB) IDs, 4LXD (Souers et al., 2013), and 6GL8 (Casara et al., 2018)] along with two structures solved by NMR spectroscopy were retrieved from the PDB [1YSW (Oltersdorf et al., 2005) and 2O2F (Bruncko et al., 2007)]. Here, it should be mentioned that BCL-2 has a region predicted to adopt an unstructured and flexible loop, which caused the protein to be insoluble (Petros et al., 2001; Bruncko et al., 2007). Hence, in NMR studies, as first suggested by Petros et al., residues 35–91 were replaced with residues 35–50 from BCL-XL, and the C-terminal end (residues 208–219) was truncated (Petros et al., 2001). The resulting chimeric protein was very soluble, while still retaining its biological activity. Moreover, a 3D structure of BCL-2 with an intact loop region would be obtained. For crystal structures, although chimeric protein was used, the unstructured loop region could not be resolved due to low electron density and the fact that the loop region was not connected. As this could cause problems during MD simulations, we took the loop conformation from the NMR structure (PDB, 1YSW) for our modeling studies. The numbering of residues was based on the crystal structure with PDB code 4LXD. The missing atoms of proteins were added, and the ions, small molecules used to aid in crystallization, and water molecules not near the cocrystallized ligand (>5 Å) were removed using the Protein Preparation module of Maestro (Sastry et al., 2013). PROPKA (Bas et al., 2008) was employed to adjust protonation states of amino acids at pH of 7.4, and finally, in order to relax the proteins, the target protein was minimized employing the OPLS2005 forcefield parameters (Banks et al., 2005). The binding pocket of BCL-2 was classified based on cocrystallized ligands, and the residues in these regions, together with water molecules, were considered in the construction of grid lattice boxes in molecular docking.

### Molecular Docking Simulations

The docking algorithms used in this study include standard precision (SP) module of Glide (Friesner et al., 2004; Halgren et al., 2004) and Induced Fit Docking (IFD) module of Maestro (Sherman et al., 2006a,b) with flexible ligand sampling. The IFD method consists of three consequent phases, including (i) docking of the compounds while the receptor is rigid; (ii) refining the complex residues within 5 Å of the ligand using Prime module (Jacobson et al., 2004); and finally, (iii) redocking of the compounds at the refined binding pocket.

### Molecular Dynamics (MD) Simulations and Molecular Mechanics/Generalized Born Surface Area (MM/GBSA) Calculations

**Figure 1:**
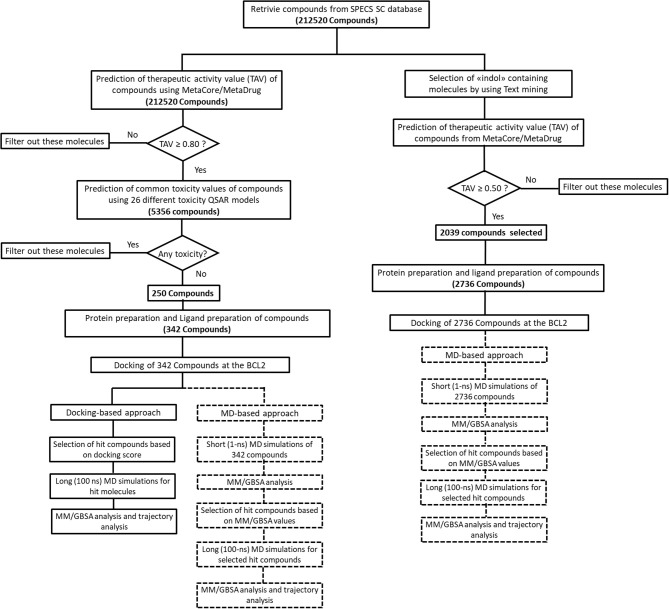
The virtual screening workflow applied with two different approaches.

We performed MD simulations for apo form of BCL-2 and complexes of BCL-2 with hit compounds using Desmond program (Bowers et al., 2006). Protein–ligand complexes were placed in the cubic boxes with explicit TIP3P water models that have 10.0 Å thickness from surfaces of protein. All systems are neutralized by adding counter ions (Na^+^ or Cl^−^ depending on the charge of the systems), and salt solution of 0.15 M NaCl was also used to adjust the concentration of the systems. The long-range electrostatic interactions were calculated by the particle mesh Ewald method (Essmann et al., 1995). A cut-off radius of 9.0 Å was used for both van der Waals and Coulombic interactions. The temperature was set as 310 K initially, and Nose–Hoover thermostat was used for adjustment (Nosé, 1984; Hoover, 1985). Martyna–Tobias–Klein protocol was employed to control the pressure, which was set at 1.01325 bar (Martyna et al., 1994). The time-step was assigned as 2.0 fs. The default values were used for minimization and equilibration steps, and finally 1-ns (for short MD simulations) and 100-ns (for long MD simulations) production run was performed for each simulation. Other details of the simulation protocols were described in our previous studies (Durdagi et al., 2016; Salmas et al., 2017; Rodrigues et al., 2018). The Prime module of Schrodinger (Jacobson et al., 2004) was used in binding free energy calculations of complexes by MM/GBSA approach (Bashford and Case, 2000). 100 trajectory frames from all MD simulation times were considered for short MD simulations. For longer MD simulations, on the other hand, 100 trajectory frames from the last half of the simulations were used for MM/GBSA calculations. OPLS3 forcefield (Banks et al., 2005) and VSGB 2.0 solvation model (Shan et al., 2011) were utilized during MM/GBSA calculations. All applied procedures for virtual screening in this study have been summarized in Figure 1.

### Time Resolved Fluorescence Resonance Energy Transfer (TR-FRET)

The BCL-2 TR-FRET Assay (CISBio, Cat. No: 79601) was used to measure the inhibition of BCL-2 to bind its ligand in the presence of BCL-2 inhibitory molecules in a homogeneous 384 well-reaction format. The assay protocol for TR-FRET analysis was performed based on the suggestions of the manufacturer. Briefly, a sample containing terbium-labeled donor, dye-labeled acceptor, BCL-2 protein, peptide ligand, and each inhibitor were incubated for 2 h. All samples and controls were studied in triplicate. Tested concentrations for the molecules were 1 nM, 10 nM, 100 nM, 1 μM, and 10 μM. The fluorescence intensity was measured using a fluorescence reader (Varioskan LUXTM, Thermo Fischer). Two sequential measurements were conducted. First, terbium-donor emission was measured at 620 nm followed by dye-acceptor emission at 665 nm; each fluorescent read was excited at 344 nm. Data analysis was performed using the TR- FRET ratio (665 nm emission/620 nm emission) value. The percent inhibitory activity of tested molecules was calculated by:

$$\begin{array}{l} \%Activity=\;\frac{FRETs-FRETneg}{FRETp-FRETneg}x100\% \end{array}$$

where *FRETs, FRETneg*, and *FRETp* are sample FRET, negative control FRET, and positive control FRET, respectively.

### Cell Culture Experiments and 3-(4 5-dimethylthiazol-2-yl)-2 5-diphenyltetrazolium bromide (MTT) Analysis

Various cancer cell lines, such as HCT-116 colon cancer, U87-MG glial tumor, and MCF7 breast cancer cell lines, were used for cell culture experiments. Cells were seeded with high glucose *Dulbecco's* Modified Eagle Medium (DMEM) medium (Biosera) supplemented with 10% fetal bovine serum (FBS) (Gibco) and 1X penicillin/streptomycin (Multicell). Twenty-four hours prior to molecule treatment, 10,000 cells were seeded into each well of 24-well-cell culture plates. Molecules were used by preparing 4 mM stock solution in dimethyl sulfoxide (DMSO) (Amresco). For molecule treatment, molecule stock solutions were diluted in DMEM with 10% FBS and added onto cells in each corresponding well. Final concentration of vehicle DMSO was 2% at maximum. Therefore, the vehicle group in experiments only included a maximum of 2% DMSO concentration. We determined the number of cells to be seeded to make sure that none of the cells reaches more than 60% confluency during the treatment period, as higher plate confluency levels would slow down cell proliferation independently from the molecule treatment.

Values of half-maximal inhibitory concentration were (IC_50_) determined by MTT cell proliferation assays. Different concentrations of molecules ranging between 10^−9^ and 10^−4^ M were tested on used cell lines with single treatment. Five hundred seventy nanometer absorbance values were recorded, and IC_50_ values were calculated by dose–response inhibition curves and non-linear regression analysis on GraphPad Prism 8 software. For cell proliferation assays, we performed 5-day experiments and repeated experiments at least three times with all cell lines. Survival rates did not change significantly after third day of treatment. Therefore, 3 days results were presented. MTT analysis was performed on 24-well plates with initially 1 × 10^4^ cells/well, grown overnight, and then treated with the selected molecules with different concentrations for at least 3 days. Following the initial incubation day, molecules were added, and, after incubation with MTT at 37°C for 4 h, formazan was solubilized with DMSO (Sigma-Aldrich, St. Louis, USA) and absorbance was measured at 570 nm.

## Results

In this work, a small molecule library (Specs-SC) that has 212,520 available drug-like compounds as well as small molecules extracted from available literature were screened initially in MC/MD platform. The molecules were filtered based on their TAV against “cancer” disease model predicted by cancer-QSAR model of MetaCore. The cancer-QSAR model was constructed with 1,053 known compounds from literature, and obtained statistical results were found as follows: Sensitivity: 0.89; Specificity: 0.83; Accuracy: 0.86; MCC: 0.72. Thus, as it can be seen, statistical results validate the constructed cancer-QSAR model. Moreover, we have selected 30 compounds that have high (IC_50_ ≤ 10 μM) inhibitory activity against BCL-2 based on biological and cell line assays to further validate used QSAR model as suggested in various previous studies (Kumar Yadav et al., 2013, 2014a,b). These molecules are also subjected to cancer-QSAR model to predict their TAV against “cancer.” Results showed that 23 out of 30 known inhibitors (more than 75% of the known inhibitors) have potential therapeutic activity (i.e., TAV ≥ 0.5) against cancer. Although the predicted QSAR values higher than 0.5 could potentially indicate therapeutic activity, in this study, we considered a higher threshold/cut-off value (≥0.8). By this way, we only considered the molecules that would be predicted as highly therapeutically active in “Cancer-QSAR” model of MC/MD. 5356 molecules had the predicted cancer therapeutic activity values equal or higher than 0.8. Compounds may show high binding affinity, but if they carry undesired side effects, they cannot be considered for future clinical studies. Therefore, their toxicity and pharmacokinetic profiles must be investigated. In our study, we used 26 different toxicity QSAR models and these diverse toxicity models cover most of the commonly observed toxicities such as cardiotoxicity, nephrotoxicity, neurotoxicity, cytotoxicity, kidney necrosis, liver necrosis, etc. Out of 5,356 identified molecules, only 250 molecules showed no toxicities in all these 26 different toxicity QSAR models. These 250 compounds were then prepared with ligand preparation module of Maestro, and, at the end, 342 structures in total, with the possible tautomeric and protonation states, were obtained. All these molecules were then used in molecular docking studies for target protein BCL-2 along with two reference molecules venetoclax and S55746 (bcl201). For S55746, the crystal structure was available; hence, after the preparation of the complex as explained in Materials and Methods section, it was subjected to MD simulations. There was no available crystal structure of BCL-2 that was cocrystallized with venetoclax when this study was conducted, though its analogs were available. Hence, venetoclax was also prepared with LigPrep and molecular docking was used to obtain complex BCL-2/venetoclax.

The common weaknesses of docking algorithms were established by the comparative evaluation study of Li et al. (2014) as mentioned above. Molecular docking method sometimes could lead to elimination of true binders and/or false positive compounds since only top-ranked molecules would be considered as selected candidates for *in vitro* tests. Protein structure being considered as mainly rigid during docking is one of the major weaknesses. As such, here we have applied two different approaches: (i) an induced fit docking in which residues in binding pocket were considered as flexible; (ii) short MD simulations in which protein–ligand complex was relaxed to dispose clashes between protein and ligand. Figure 1 shows the workflow applied in this study. It can be seen that we have also identified compounds that contain “*indol*” phrases using text mining to be considered for ligand- and structure-based studies of BCL-2 inhibitors.

### Docking-Based Approach for Selection of Hit Molecules

The top-docking scores of five molecules and their 2D structures as well as corresponding data for reference molecules venetoclax and S55746 could be found in Table S1. To test the validity and reliability of docking approach as performed in previous studies (Chen et al., 2006), we have also redocked the cocrystallized ligand found in the crystal structure of BCL-2 (PDB ID, 4LXD). We have seen that the docking pose obtained with our protocol was able to reproduce the crystal pose with a root mean square deviation (RMSD) of 0.65 Å (i.e., after alignment between docked pose and cocrystallized pose, RMSD was 0.65 Å). As it can be seen from Table S1, venetoclax has the highest docking score (−15.46 kcal/mol) at BCL-2 cavity. However, venetoclax is a very large molecule [molecular weight (MW), 868 g/mol] that contains 61 non-hydrogen atoms, which could lead to high docking score, and in fact its ligand efficiency score (i.e., docking score per number of non-hydrogen atoms) was lower than the suggested compounds (Table S1). The molecules with the top-docking scores were smaller than venetoclax and S55746. However, they have high ligand efficiency scores, which could indicate that they could be lead compounds for further studies. For that reason, we performed MD simulations (100 ns) for these compounds in complex with BCL-2 protein starting from IFD docking poses. Although we carried out MD simulations for all five complexes as well as for two reference molecules in complex with BCL-2, we will only discuss the results for three compounds: **43** (Specs ID: AO-081/41887762); compound **58** (Specs ID: AJ-292/12931005); and compound **243** (Specs ID: AN-698/40780701), as they were chosen for *in vitro* studies.

### MD-Based Approach for the Selection of Hit Molecules

The top-scoring docking poses for all 342 compounds were subjected to 1-ns short MD simulations, and the average binding free energies were calculated for MD trajectory frames using MM/GBSA approach. An in-house script was used for the preparation of simulation boxes as well as for the analysis of MD simulations. Compounds were then ranked based on average MM/GBSA scores, and, as shown in Figure S1, the normal distribution of MM/GBSA scores of studied 342 compounds and Z-scores of the distribution curves were plotted. Then, we selected compounds that have average MM/GBSA values above Z ≤ −2, i.e., 12 molecules were chosen. The complexes of these compounds with BCL-2 were subjected to longer (100 ns) MD simulations. The structures and average MM/GBSA values of all these selected compounds could be found in Table S2. Although we have performed longer MD simulations for all 12 compounds in complex with the target protein, we selected only three of the compounds for *in vitro* tests, **258** (Specs ID: AK-968/12163470), **292** (Specs ID: AK-968/11842328), and **243** (Specs ID: AN-698/40780701). It should be noted that compound **243** was also found as a hit compound and selected based on docking approach.

### Text Mining Approach for Selection of Hit Compounds

**Figure 2:**
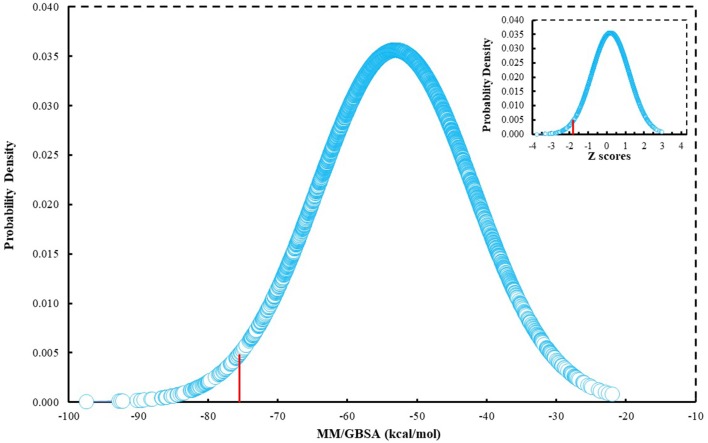
MM/GBSA scores of around 2700 molecules that include “indol” phrases.

Since many currently used FDA-approved chemotherapeutics include indole derivatives, Specs-SC database that includes only “*indol”* groups (i.e., indoles, indolons, bisindoles, etc.) were also screened at the binding pocket of the BCL-2. Thus, “*indol”* keyword was searched as text within the 212,000 compounds and around 2700 compounds were identified. These “*indol”* phrase containing molecules were subjected to binary QSAR tests using MC/MD platform and specifically the “cancer-QSAR” model was chosen as before. Since indole derivatives are known to have high therapeutic activity potential, here we initially used a lower TAV threshold (0.5) to begin screening with a large number of molecules that include the “*indol”* phrase. Molecules that showed higher TAV values than 0.5 were docked at the binding pocket of BCL-2 using Glide/SP. Top-docking poses of these compounds were then used in MD simulations. 2700 individual MD simulations boxes were prepared with an in-house script, and 1-ns MD simulations were conducted and the average MM/GBSA scores were calculated. The normal distribution of MM/GBSA values as well as *Z*-scores of the distribution showed that there were 83 compounds with Z-scores lower than −2 (see Figure 2). As performing 100-ns MD simulation for all 83 complexes would require considerable computer time and power, we instead chose to perform 10-ns MD simulations for these compounds in complex with BCL-2 and again used MM/GBSA approach to calculate their average binding free energies. After 10-ns MD simulations, top-10 MM/GBSA-scored “*indol”* phrase containing molecules were forwarded for 100-ns MD simulations and their average MM/GBSA scores were calculated. Table S3 shows the 2D structures and average MM/GBSA scores for these 10 compounds. We have selected two of them for *in vitro* studies: **ind-199** (AG-205/12549135) **and ind-435** (AN-329/13484046).

### Analysis of Selected Compounds and Their Interactions With BCL-2

**Figure 3:**
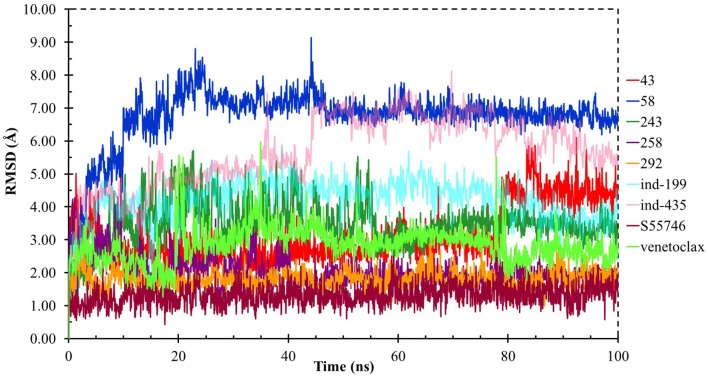
ProFit RMSD graphs for the hit and reference compounds.

**Figure 4:**
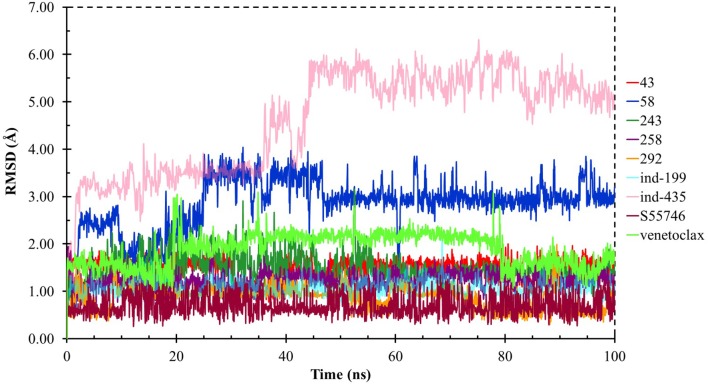
LigFit RMSD graphs for the hit and reference compounds.

Although molecular docking studies could give an initial insight into protein–ligand interactions, it is always crucial to understand the maintenance of these interactions and perform dynamical studies as MD simulations for complexes. Hence, we performed MD simulations and analyzed the interactions observed during the simulations between protein and ligands. While we conducted 100-ns MD simulations for 29 compounds in total (including the reference molecules) in complex with BCL-2, we selected seven of them for *in vitro* studies based on their docking scores, MM/GBSA values, and their interactions with the target protein. Here, we will focus our analysis and discussion on these seven compounds that could be lead compounds as BCL-2 inhibitors. Before analyzing the ligand–protein interactions, the trajectories obtained from the simulations were firstly analyzed to examine the protein and ligand structure stability. RMSD and the root mean square fluctuations (RMSF) were used to measure the displacements of atoms for each frame with respect to the initial frame/structure and to categorize the local changes along protein structures, respectively (Figures S2, S3). Here, we have only plotted the RMSD graphs of studied proteins based on alpha carbons (C_α_). As it can be seen from the figure, for compounds other than “*indol”* phrase containing ones, the RMSD plots do not change significantly after 50-ns and they reach a plateau. However, mainly in indol-containing molecules, RMSD values did not stabilize and conformational changes were observed during MD simulations (Figure S4). It can be seen that it was the unstructured loop region 31–89, not alpha-helix regions, that had higher displacement (Figures S3, S4). For **ind-199**, some unexpected helix formation is observed for this region, though the helix could not be conserved. The RMSF plot for protein targets in complex with selected compounds also confirmed that it was the loop region for which highest displacements were observed (Figure S3). Additionally, we checked the RMSD of the ligand molecules by considering two different fitting modes: “fit on protein/profit” and “fit on ligand/ligfit.” While the first mode indicates the structural stability of ligand with respect to protein, i.e., its translational motion, the second mode shows the internal fluctuations of the ligand atoms in its binding pocket, i.e., its rotational motion. As can been seen from Figure 3, the profit RMSD plot shows that after initial 50 ns, most of the compounds did not move away from the binding pocket. However, compound **58** had a very high RMSD value (around 7.0 Å), which showed that it has high mobility in the binding pocket. In fact, Figure S4 shows that the compound **58** completely changed its initial binding pose in the hydrophobic groove just after initial 20 ns, but then it did not have high mobility and stayed in the pocket as can be seen by lower RMSD values and also small conformational changes. Indol-containing molecule **ind-435** also displayed high mobility in the binding pocket as can be observed in Figure 3 and Figure S4. The RMSD for this compound did not really reach a plateau value. The size of this compound was actually considerably bigger than other molecules and has flexible regions. Hence, it extended from its initial binding pocket to the one next to it (e.g., hydrophobic grooves P1 to P4). Compound **243** has also higher profit RMSD values especially after 80 ns, and careful analysis of MD trajectories showed that it was mostly the phenyl ring that was attached thiazolidine group moving in pocket P1. The rotational movements (ligfit RMSDs) of the selected compounds could be seen in Figure 4. We observed that venetoclax did not also obtain a stable RMSD plot, though the values themselves were not higher than 3.0 Å for ligfit mode. As a large molecule, these rotational movements were not surprising. It was also not unexpected for **ind-435** as a large molecule with flexible alkyl chain to have high rotational RMSD values as seen in Figure 4. Compound **58** was found with higher RMSD values for ligfit mode; however, after 50 ns, the changes in RMSD values were smaller. All of the compounds at the end reached a kind of plateau value for rotational RMSD of ligands.

**Figure 5:**
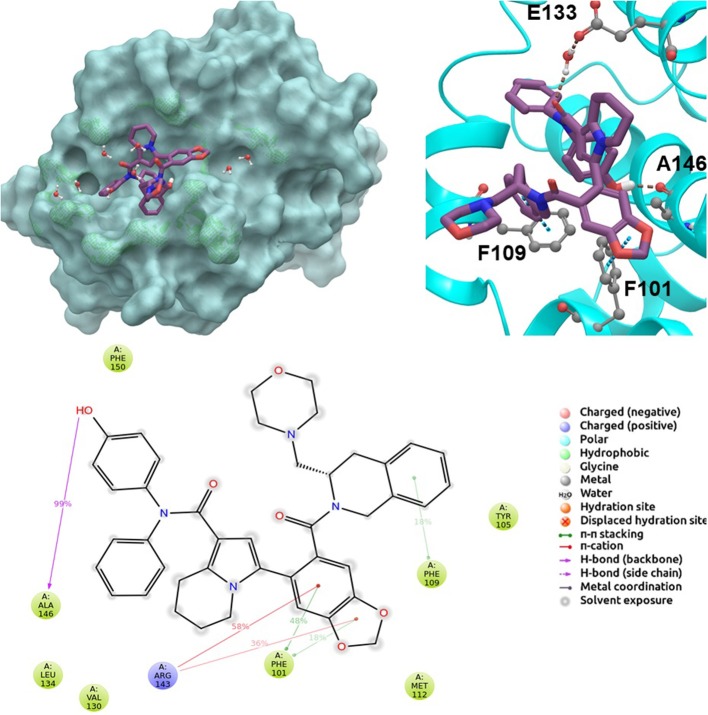
2D and 3D ligand interactions diagrams of selected positive control molecule S55746 at the binding pocket of BCL-2. Surface and ribbon representation are displayed for representative structure obtained from MD simulations, while 2D interaction diagram shows the systematic details of protein-ligand interactions observed during MD.

**Figure 6:**
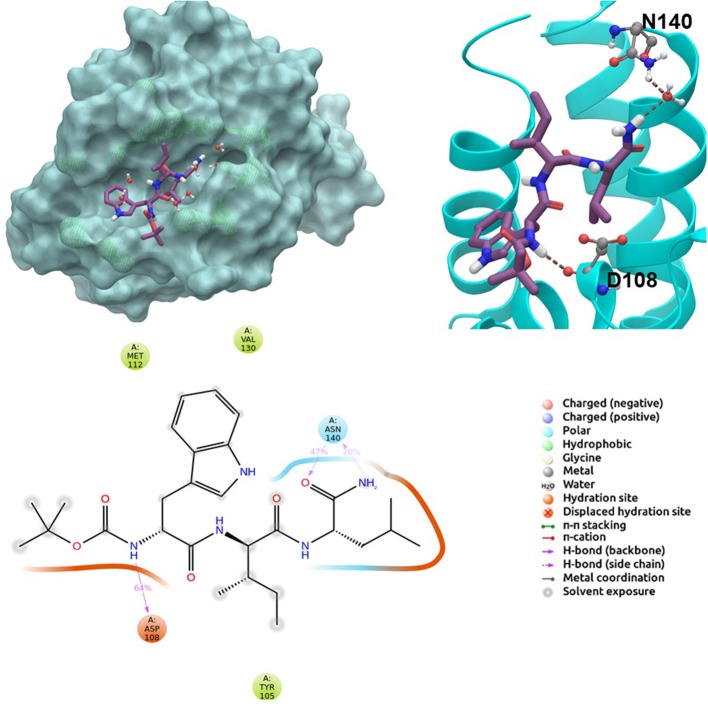
2D and 3D ligand interactions diagrams of selected hit molecule **58** at the binding pocket of BCL-2. Surface and ribbon representation are displayed for representative structure obtained from MD simulations, while 2D interaction diagram shows the systematic details of protein-ligand interactions observed during MD.

BCL-2 protein interacts with BH3-only proteins via hydrophobic groove on its surface, which contains four pockets: P1, P2, P3, and P4 pockets. To prevent the interaction of proapoptotic proteins such as BAX and BAK with BCL-2, which is an antiapoptotic protein, these pockets need to be filled by either small molecules or BH3-mimetics. It is important for these molecules to interact with key residues that mediate interaction between BCL-2 and BH3-only proteins. Based on the literature data, some of these crucial residues are as follows (numbering based on PDB code 4LXD): Asp100, Phe101, Arg104, Tyr105, Asp108, Phe109, Tyr199, Asn140, Gly142, Arg143, and Ala146. Additionally, we have analyzed the MD simulations of reference compounds venetoclax and S55746 in complex with BCL-2. The protein surfaces as well as 2D and 3D ligand interactions between protein and molecules were represented for venetoclax and S55746 in Figure 5 and Figure S5, respectively. Consistent with the previously published data, S55746 bind and fill the pockets P1 to P2, while venetoclax could fill all four pockets on the surface from P1 to P4 (Birkinshaw et al., 2019). When this project was initiated, there was no cocrystallized venetoclax-bound form of the BCL-2. Here, we also checked the binding pose of venetoclax as the crystal structure of BCL-2 bound to venetoclax recently became available (Birkinshaw et al., 2019). A slight difference in binding pose of venetoclax was in the more flexible region of oxane fragment; however, with the rest of the compound, similar amino acid moieties interact by the identical parts of venetoclax in both poses. Based on the trajectory analysis of venetoclax, it was seen that venetoclax preserved its interactions with Asp100 and Phe101 more than 50% of the simulation time (Figure S5). Gly142 and Arg104 were also seen as interacting residues. Interaction with the backbone carbonyl oxygen atom of Ala143 was the most conserved interaction during MD simulations for S55746 (99%, Figure 5). Also, Arg143 and Phe101 formed π-cation and π-π stacking interactions with S55746, respectively. Based on these results, we analyzed the complexes of BCL-2 protein with selected hit compounds as well as examined their ability to fill the pockets P1 to P4. Compound **43**, which can mainly bind BCL-2 via P2 and P3 pockets, consistently formed hydrogen bonds with Asp137 (96%) and π-π stacking interactions with residues Phe101 and Tyr105 of P2 pocket, although these later interactions were not preserved (16 and 24% of MD time, Figure S6). Compound **58** can mainly fill the pockets P1 to P3 of BCL-2 similar to S55746. It formed stable hydrogen bonding interactions with two key residues Asp108 and Asn140 (64 and 67%, respectively, Figure 6). Compound **243**, on the other hand, interacted with the residues of pockets P1 to P4. A π-π stacking interaction with Phe101 of P1 pocket was observed for 49% of MD simulation time, while its interactions with other key residues such as Tyr105, Asn140, and Arg143 were less conserved (Figure S7). Compound **258** was mainly found in pockets P2 to P4, and its most conserved interaction was observed to be with Tyr105. Not only did it form hydrogen bonds with Tyr105 but it also formed π-π stacking interactions, albeit they were not persistent interactions (17%, Figure S8). Compound **292** was an analog of compound **258;** as such, similar binding poses were expected, but their binding modes were quite different. Compound **292** formed persistent hydrogen bonding interactions with Asp108 (73%, Figure S9). H-bond and π-cation interactions with Arg143 were also observed for compound **292**. The chosen “*indol”* containing molecules were larger molecules compared to other five selected molecules; as such, they are able to fill the pockets P1 to P4. Compound **ind-199** formed a stable hydrogen bond with Asn140 (70%), π-cation, and salt bridge interactions with Arg104 (Figure S10). Although it also formed π-π stacking interactions with Phe109 and Phe150, these interactions were not persistent (13 and 22%, respectively). Compound **ind-435** not only filled all four pockets but also moved closer to carbonyl terminal part of BCL-2. A persistent hydrogen bond with Asp100 (55% of MD time) was observed, and additionally it interacts and with residues Phe100 and Arg143 (Figure S11).

**Figure 7:**
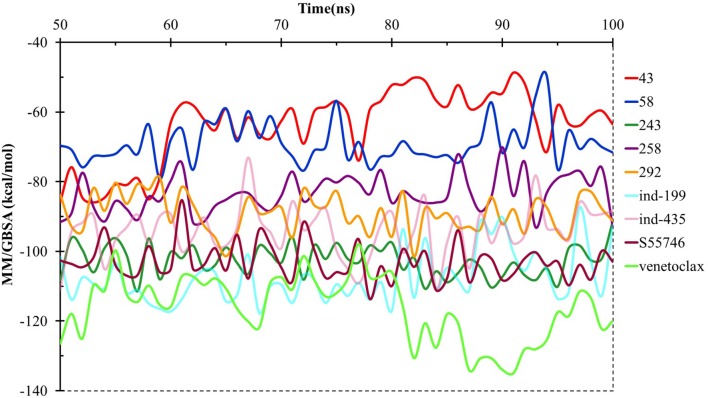
MM/GBSA free energy analysis for the studied molecules at the binding pocket of BCL-2 throughout the last half of the MD simulations.

We have also calculated the binding free energies for selected compounds as well as reference molecules in complex with BCL-2 using MM/GBSA approach after 100 ns MD simulations. In Figure 7, the MM/GBSA energies calculated for the trajectory frames observed during MD were plotted. It can be seen that venetoclax had lower MM/GBSA values compared to selected compounds. However, some of the selected compounds such as compound **243**, **ind-435**, and **ind-199** have considerable MM/GBSA values to other reference molecule S55746.

### TR-FRET Analysis Confirms the Inhibitory Activity of Identified Hit Molecules on BCL2

**Figure 8:**
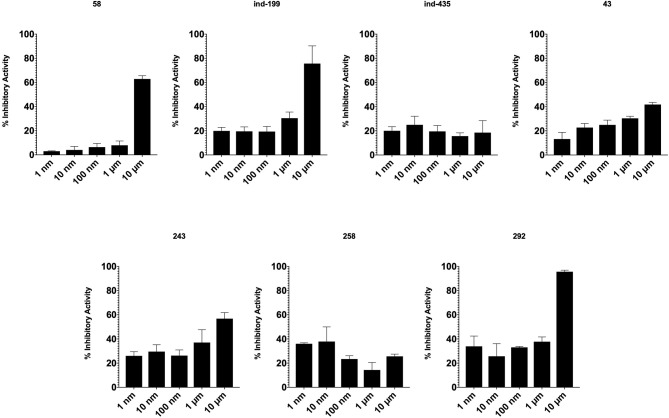
Inhibitory activity of tested molecules. Activity of the molecules was assessed by the formula given in Materials and Methods section.

TR-FRET analysis revealed that four of seven tested molecules compete with BCL2 ligand in binding. In presence of inhibitory molecules, BCL2 binding to its ligand was suppressed in a concentration- dependent manner. Compounds **58**, **ind-199**, **243**, and **292** showed the maximum inhibitory effect in ranging between 60 and 100% in 10 μM concentrations (Figure 8). However, three of selected molecules showed minimal inhibitory activity on BCL2 ranging from 10 to 40% with concentration-independent manner. We suppose that activity is correlated with solubility of the molecules since inactive molecules were found among partially soluble molecules.

### Cell Proliferation Was Restricted by Using BCL2 Inhibitory Molecules

Together with TR-FRET analysis, selected hit compounds were also evaluated in various cancer cell lines, such as HCT-116 colon cancer, U87-MG glial tumor, MCF7 breast cancer cell lines, and IC_50_ values of selected molecules were calculated (Table 1). Of seven selected hits, five of them showed micromolar level of inhibitory concentrations, which is an acceptable range in cell culture experiments. Compound **258** did not show inhibitory activity on any of the tested cell-line assays.

**Table 1:** The specs ID, 2D structure, average MM/GBSA values, and maximal % inhibitory activity at 10μM concentrations, as well as IC_50_ values of selected hit compounds and reference molecules.

**Compounds (specs ID)**	**2D structure**	**MM/GBSA (kcal/mol)**	**% inhibitory activity at 10 μM**	**IC_**50**_ (μM) for HTC116 cells**	**IC_**50**_ (μM) for U87-MG cells**	**IC_**50**_ (μM) for MCF7 cells**
**43** (AO-081/41887762)	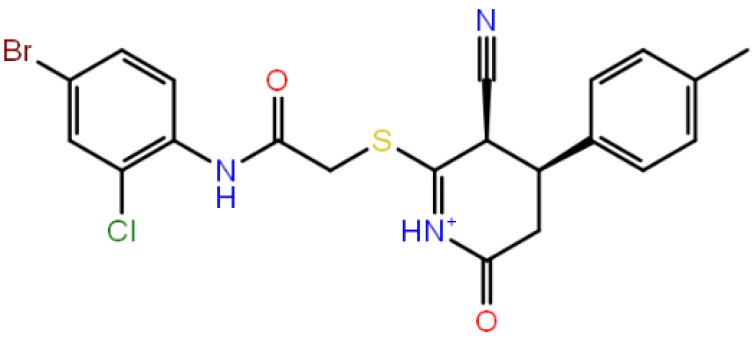	−64.15 ± 10.37	41.78 ± 1.76	24 ± 2.53	26 ± 1.73	30 ± 8.27
**58 (**AJ-292/12931005)	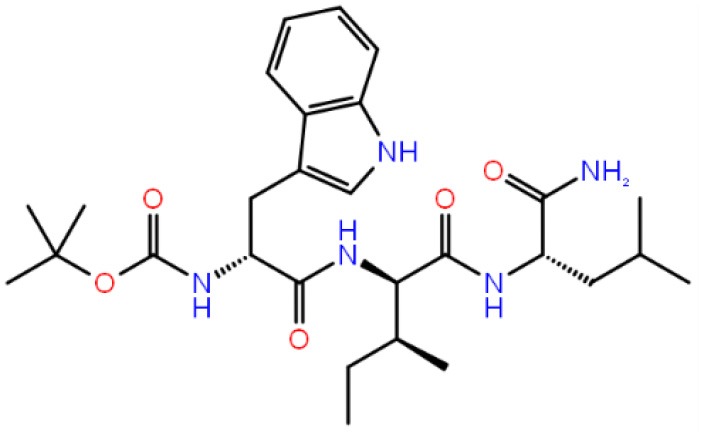	−68.84 ± 6.03	62.84 ± 2.73	17 ± 1.59	18 ± 2.29	21 ± 3.34
**243 (**AN-698/40780701**)**	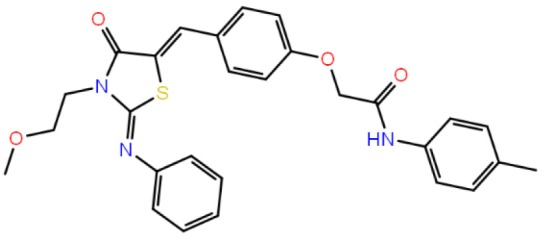	−102.65 ± 4.56	56.78 ± 5.02	25 ± 1.77	29 ± 2.55	31 ± 3.96
**258** (AK-968/12163470)	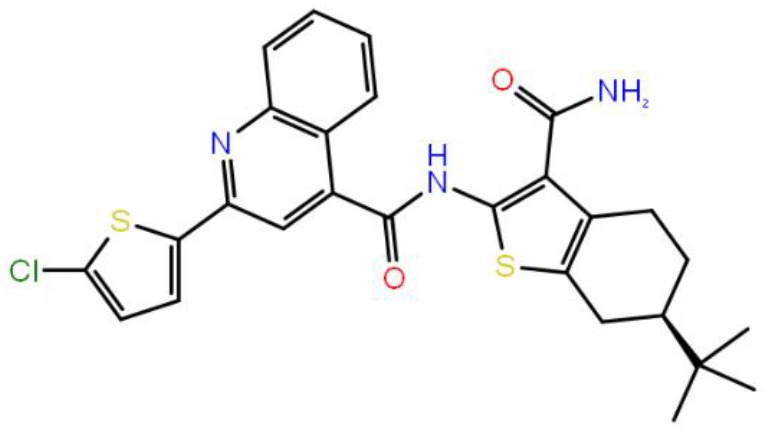	−83.50 ± 5.46	25.60 ± 1.84	NA^†^	NA^†^	NA^†^
**292** (AK-968/11842328)	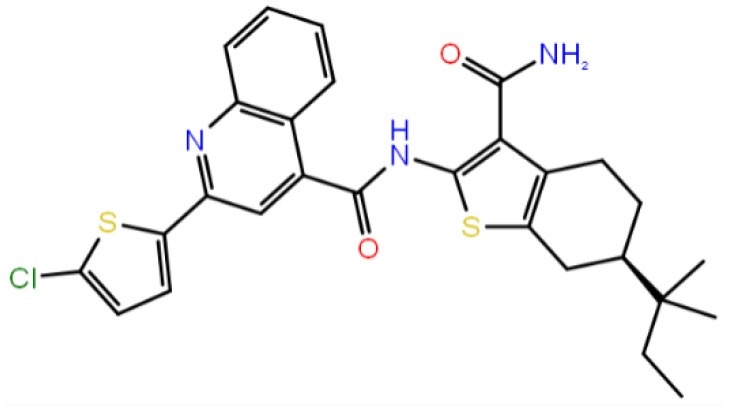	−88.93 ± 5.46	95.63 ± 1.27	130 ± 25.59	122 ± 33.21	150 ± 33.50
**ind-199 (AG-205/12549135)**	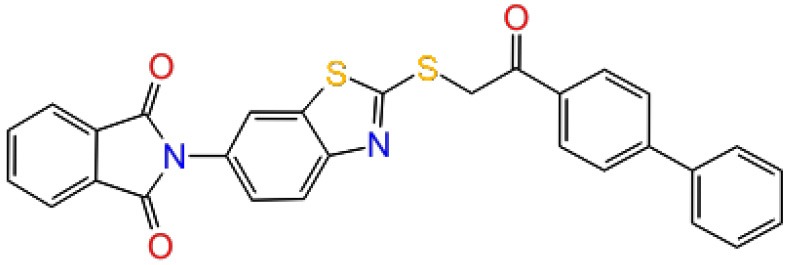	−107.41 ± 7.49	75.64 ± 14.65	22 ± 3.41	28 ± 4.46	26 ± 4.89
**ind-435** (AN-329/13484046)	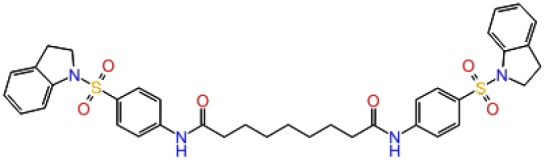	−94.17 ± 7.28	18.51 ± 9.91	NA^†^	NA^†^	NA^†^
venetoclax	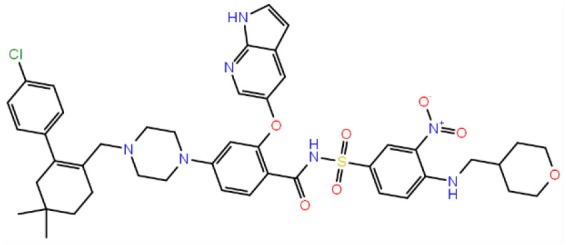	−116.31 ± 9.20	NA^†^	3.5^*^	22.9^*^	24.7^*^
S55746 (CHEMBL3958369)	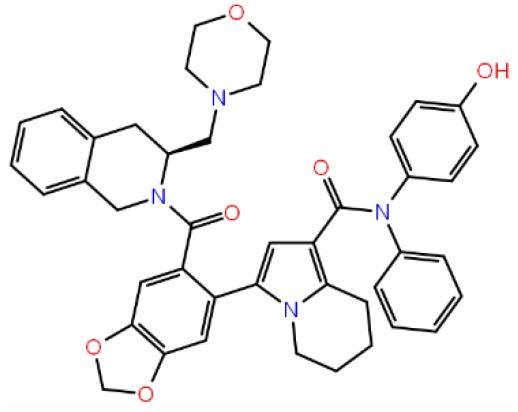	−101.43 ± 4.20	NA^†^	NA^†^	NA^†^	NA^†^

† *Not applied*.

* *Taken from The Genomics of Drug Sensitivity in Cancer (GDSC) database (www.cancerRxgene.org) for HTC-116 cell line*.

**Figure 9:**
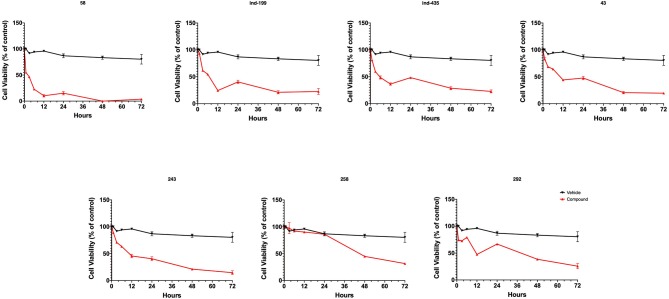
MTT cell proliferation assay. Molecules having inhibitory activity were shown. Tested concentration for each drug was 100 μM. Statistical significance in graphs was determined by comparing each treatment group with DMSO control using ANOVA testing, and significance is considered as *p* < 0.001. Error bars show standard deviation.

To test whether BCL-2 inhibitory molecules had any effect on biological activity, we conducted cell proliferation assays and also evaluated apoptosis by observing cell structure and counting apoptotic cells. All molecules were tested on three different cancer cell lines, and all molecules showed similar effects on different cell types. The dose–response curves for all cell lines were shown in Figures S12–S14. Of seven molecules, four showed biological activity on MTT experiments. Molecules **58**, **ind-199**, **43**, and **243** with 100 μM concentration significantly limited the cell proliferation capacity of cancer cells. Four biologically active molecules showed their efficacy starting from the first hour of treatment by decreasing the number of proliferating cells. At the first day of treatment, only 60–70% of cells survived, while the number of proliferating cells decreased to <40% at the end of third day (Figure 9). MTT assay results for cell lines U87-MG and MCF7 were displayed in Figures S15, S16, respectively. Compared to untreated and DMSO only treated (vehicle) group, these four hit molecules showed significant activity. Lack of activity of other three compounds might be due to their low/moderate solubilities. Furthermore, the activity of the molecules was more dominant at cancer cells. We also tested these molecules on non-cancerous HUVEC cells, and none of the molecules showed significant reduction in cell viability (Figure S17). When we observed the cells under a microscope for the inactive molecules, we saw precipitates of molecules. Further, despite all efforts for increasing the solubility levels of these inactive compounds in DMSO, it failed to obtain a pure solubilized form of the molecules.

**Figure 10:**
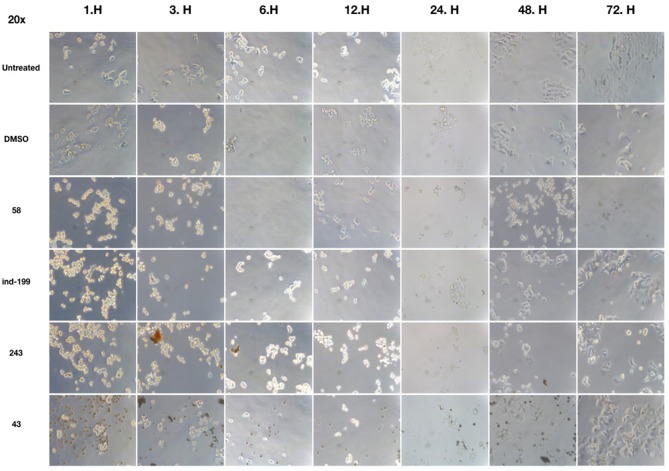
Microscopic evaluation of HCT-116 cells for compounds **58** (AJ-292/12931005), **ind-199** (AG-205/12549135), **43** (AO-081/41887762), and **243** (AN-698/40780701). Cells were photographed and observed under microscope for 3 days. Vehicle group showed neat proliferation of cells as untreated group did, whereas molecule-treated groups reduced proliferation and showed apoptotic cell structures. Compound **58** (AJ-292/12931005) showed clear apoptotic activity from the first day, whereas other molecules showed limited activity. At 48 and 72 h of treatment in other groups, resistant cells are visible together with the unresolved molecule precipitates. However, there are no resistant cells and all cells seemed to be affected by the molecule **58** on treated group for all 3 days.

We also observed massive cell detachment and dying cells in cell culture plates (Figure 10), and formation of apoptotic cell bodies having circular structure rather than normal. Especially for the compound **58**, apoptotic cell bodies were abundant, and it immediately affected the cells upon the first hour of treatment. However, for the compounds **43** and **243**, despite apoptotic cell bodies having formed, there were still some unaffected cell residues that survived and proliferated. Cell detachment, cell death, and apoptotic bodies indicate apoptotic cell death. Our data altogether suggest that at 100 μM, concentrations of compounds **58**, **ind-199**, **43**, and **243** induce apoptotic cell death.

Inhibition of antiapoptotic protein BCL-2 that is overexpressed in cancer cells is one of the most studied approaches in cancer research. Currently, venetoclax is the only approved drug by the FDA for the treatment of chronic lymphocytic leukemia (CLL), and it is a selective BCL-2 protein inhibitor. Although it has a very high affinity for BCL-2 as shown in various studies performed on different cancer cell lines (Yang et al., 2012) (www.cancerrxgene.org), resistance to this drug has already been observed (Birkinshaw et al., 2019). Hence, it is necessary to suggest new compounds and scaffolds as BCL-2 inhibitors that could be more efficient against mutations on the target structure and have no side effects. As such, in this study, we performed combined ligand- and structure-based approaches as well as text mining to propose new inhibitors against BCL-2 target protein. We selected seven hit compounds of which two are “*indol*”-based molecules. These compounds were considered in cancer cell line assays, and their IC_50_ values are calculated (Table 1). Based on the experimental findings, compound **58**, which has an IC_50_ value of 17 μM, was found to promote apoptosis, and it was the most effective of the seven compounds. Some of the selected hit molecules (compounds **258** and **292**) for *in vitro* analysis could only be partially solubilized in DMSO; therefore, solubility issue may restrict the activity of the compounds.

When we have considered and compared the structures of selected hit molecules, specifically compounds that inhibited the proliferation of cancer cells such as compounds **58**, **ind-199**, **43**, and **243**, we have observed that all except compound **58** contains sulfur-containing groups such as sulfanyl or thiazole derivatives. All four molecules contain aromatic rings as well as amide groups in their structures, which are also groups observed in FDA-approved drug venetoclax and under development compound S55746. It must be noted that together with identified two indol-based compounds, **58** also involves an indole ring in its structure; thus, it validates the importance of indoles or indole derivatives in the scaffolds of potent BCL2 inhibitors. It must also be noted that the proposed hit molecules have lower molecular weights (MW) (between 490 and 529 g/mol) than the known BCL-2 inhibitors venetoclax (MW, 868 g/mol) and S55746 (MW, 710 g/mol) which indicate that the suggested compounds could be used as lead compounds for further optimization studies by small modifications. See .sdf file for structures of hit compounds along with their properties such as TAV, docking score, toxicity values, etc. in Data Sheet 1 in the Supplementary Material.

Overall, our results surprisingly show that docking-initiated screening has a better success rate compared to MD-initiated screening. However, this surprising result may be due to unexpected partial solubilities of some of the tested compounds that showed limited activity on cells.

## Conclusions

In this work, a molecular library (Specs-SC) composed of 212,520 molecules was first filtered for their therapeutic effect against cancer, and then obtained molecules again filtered to remove toxic compounds using MC/MD from Clarivate Analytics. Identified 342 non-toxic and potent compounds using MC/MD were then screened based on target-driven approaches using available BCL-2 structures. In order to compare the both structural and energetic results, known BCL-2 inhibitors were used as positive control molecules and same computational protocols were applied for these compounds. Identified hit molecules from both docking and short (i.e., 1-ns) MM/GBSA calculations that have similar/better binding energies were compared to known inhibitors, then subjected to longer (i.e., 100-ns) MD simulations. In the virtual screening, two different strategies were considered and compared in the selection of hit compounds: (i) Compounds were ranked by their docking scores and long (100-ns) MD simulations were performed for the selected compounds and average MM/GBSA energies were calculated; (ii) Short (1-ns) MD simulations were performed for top-docking poses of all 342 compounds and average MM/GBSA scores were considered for the selection of molecules in long (100-ns) MD simulations of small molecules database. At the end, seven molecules were suggested as new scaffolds for inhibition of BCL-2. Compounds **58** (AJ-292/12931005), **ind-199** (AG-205/12549135), **43** (AO-081/41887762), and **243** (AN-698/40780701) with 100 μM concentration significantly limited the cell proliferation capacity of cancer cells. Four biologically active molecules showed their efficacy starting from the first hour of treatment by decreasing the number of proliferating cells. At the first day of treatment, only 60–70% of cells survived, while the number of proliferating cells decreased to <40% at the end of third day. TR-FRET analysis revealed that hit compounds **58**, **ind-199**, **243**, and **292** showed the maximum inhibitory effect ranging between 60 and 100% in 10 μM concentration. Thus, most of the active compounds found in the cell line tests were also found potent in enzymatic assays. Results showed that compounds identified via integrated text-mining and docking initiated MM/GBSA-scores based approach has higher success rate. Thus, the results of this study may open new avenues for the designing of new BCL-2 inhibitor scaffolds.

## Data Availability Statement

The raw data supporting the conclusions of this article will be made available by the authors, without undue reservation, to any qualified researcher.

## Author Contributions

BD and SD constructed the methodology and wrote the manuscript. SD also guided the studies, revised the paper, and managed laboratory resources and research funding. GT and BD performed molecular modeling studies. MO, SC, and TA conducted the experimental studies and wrote the manuscript.

### Conflict of Interest

The authors declare that the research was conducted in the absence of any commercial or financial relationships that could be construed as a potential conflict of interest.

## References

[B1] Banks J. L. Beard H. S. Cao Y. Cho A. E. Damm W. Farid R.. (2005). Integrated modeling program, applied chemical theory (IMPACT). J. Comput. Chem. 26, 1752–1780. 10.1002/jcc.20292 16211539 PMC2742605

[B2] Bas D. C. Rogers D. M. Jensen J. H. (2008). Very fast prediction and rationalization of pKa values for protein–ligand complexes. Proteins 73, 765–783. 10.1002/prot.22102 18498103

[B3] Bashford D. Case D. A. (2000). Generalized born models of macromolecular solvation effects. Annu. Rev. Phys. Chem. 51, 129–152. 10.1146/annurev.physchem.51.1.129 11031278

[B4] Billard C. (2013). BH3 mimetics: status of the field and new developments. Mol. Cancer Ther. 12, 1691–1700. 10.1158/1535-7163.MCT-13-0058 23974697

[B5] Birkinshaw R. W. Gong J. N. Luo C. S. Lio D. White C. A. Anderson M. A.. (2019). Structures of BCL-2 in complex with venetoclax reveal the molecular basis of resistance mutations. Nat. Commun. 10:2385. 10.1038/s41467-019-10363-1 31160589 PMC6547681

[B6] Bissantz C. Folkers G. Rognan D. (2000). Protein-based virtual screening of chemical databases. 1. Evaluation of different docking/scoring combinations. J. Med. Chem. 43, 4759–4767. 10.1021/jm001044l 11123984

[B7] Bowers K. J. Chow D. E. Xu H. Dror R. O. Eastwood M. P. Gregersen B. A. (2006). Scalable algorithms for molecular dynamics simulations on commodity clusters, Paper Presented at the SC'06: Proceedings of the 2006 ACM/IEEE Conference on Supercomputing (Tampa, FL). 10.1145/1188455.1188544

[B8] Bruncko M. Oost T. K. Belli B. A. Ding H. Joseph M. K. Kunzer A.. (2007). Studies leading to potent, dual inhibitors of Bcl-2 and Bcl-xL. J. Med. Chem. 50, 641–662. 10.1021/jm061152t 17256834

[B9] Bursulaya B. D. Totrov M. Abagyan R. Brooks C. L. (2003). Comparative study of several algorithms for flexible ligand docking. J. Comput. Aided Mol. Des. 17, 755–763. 10.1023/B:JCAM.0000017496.76572.6f 15072435

[B10] Casara P. Davidson J. Claperon A. Le Toumelin-Braizat G. Vogler M. Bruno A.. (2018). S55746 is a novel orally active BCL-2 selective and potent inhibitor that impairs hematological tumor growth. Oncotarget 9, 20075–20088. 10.18632/oncotarget.24744 29732004 PMC5929447

[B11] Chen H. Lyne P. D. Giordanetto F. Lovell T. Li J. (2006). On evaluating molecular-docking methods for pose prediction and enrichment factors. J. Chem. Inf. Model 46, 401–415. 10.1021/ci0503255 16426074

[B12] Chiba S. Ishida T. Ikeda K. Mochizuki M. Teramoto R. Taguchi Y. H.. (2017). An iterative compound screening contest method for identifying target protein inhibitors using the tyrosine-protein kinase yes. Sci. Rep. 7:12038. 10.1038/s41598-017-10275-4 28931921 PMC5607274

[B13] Chung C. (2018). Restoring the switch for cancer cell death: targeting the apoptosis signaling pathway. Am. J. Health Syst. Pharm. 75, 945–952. 10.2146/ajhp170607 29759975

[B14] Cory S. Huang D. C. Adams J. M. (2003). The Bcl-2 family: roles in cell survival and oncogenesis. Oncogene 22, 8590–8607. 10.1038/sj.onc.1207102 14634621

[B15] Cross J. B. Thompson D. C. Rai B. K. Baber J. C. Fan K. Y. Hu Y.. (2009). Comparison of several molecular docking programs: pose prediction and virtual screening accuracy. J. Chem. Inf. Model. 49, 1455–1474. 10.1021/ci900056c 19476350

[B16] Dewson G. Kluck R. (2010). Bcl-2 family-regulated apoptosis in health and disease. Cell Health Cytoskeleton 2, 9–22. 10.2147/CHC.S6228

[B17] Durdagi S. Aksoydan B. Erol I. Kantarcioglu I. Ergun Y. Bulut G.. (2018a). Integration of multi-scale molecular modeling approaches with experiments for the *in silico* guided design and discovery of novel herg-neutral antihypertensive oxazalone and imidazolone derivatives and analysis of their potential restrictive effects on cell proliferation. Eur. J. Med. Chem. 145, 273–290. 10.1016/j.ejmech.2017.12.021 29329002

[B18] Durdagi S. Dogan B. Erol I. Kayik G. Aksoydan B. (2019). Current status of multiscale simulations on GPCRs. Curr. Opin. Struct. Biol. 55, 93–103. 10.1016/j.sbi.2019.02.013 31082696

[B19] Durdagi S. Salmas R. E. Stein M. Yurtsever M. Seeman P. (2016). Binding interactions of dopamine and apomorphine in D2High and D2Low states of human dopamine D2 receptor using computational and experimental techniques. ACS Chem. Neurosci. 7, 185–195. 10.1021/acschemneuro.5b00271 26645629

[B20] Durdagi S. Tahirul Qamar M. Salmas R. E. Tariq Q. Anwar F. Ashfaq U. A. (2018b). Investigating the molecular mechanism of staphylococcal DNA gyrase inhibitors: a combined ligand-based and structure-based resources pipeline. J. Mol. Graph. Modell. 85, 122–129. 10.1016/j.jmgm.2018.07.010 30176384

[B21] Eimon P. M. Ashkenazi A. (2010). The zebrafish as a model organism for the study of apoptosis. Apoptosis 15, 331–349. 10.1007/s10495-009-0432-9 20033783

[B22] Erol I. Cosut B. Durdagi S. (2019). Toward understanding the impact of dimerization interfaces in angiotensin II type 1 receptor. J. Chem. Inf. Model. 59, 4314–4327. 10.1021/acs.jcim.9b00294 31429557

[B23] Essmann U. Perera L. Berkowitz M. L. Darden T. Lee H. Pedersen L. G. (1995). A smooth particle mesh Ewald method. J. Chem. Phys. 103, 8577–8593. 10.1063/1.470117

[B24] Fesik S. W. (2005). Promoting apoptosis as a strategy for cancer drug discovery. Nat. Rev. Cancer 5, 876–885. 10.1038/nrc1736 16239906

[B25] Friesner R. A. Banks J. L. Murphy R. B. Halgren T. A. Klicic J. J. Mainz D. T.. (2004). Glide: a new approach for rapid, accurate docking and scoring. 1. Method and assessment of docking accuracy. J. Med. Chem. 47, 1739–1749. 10.1021/jm0306430 15027865

[B26] Fu Y. Sun Y.-N. Yi K.-H. Li M.-Q. Cao H.-F. Li J.-Z.. (2018). Combination of virtual screening protocol by *in silico* toward the discovery of novel 4-hydroxyphenylpyruvate dioxygenase inhibitors. Front. Chem. 6:14. 10.3389/fchem.2018.00014 29468151 PMC5807903

[B27] Gandhi L. Camidge D. R. De Oliveira M. R. Bonomi P. Gandara D. Khaira D.. (2011). Phase I study of navitoclax (ABT-263), a novel Bcl-2 family inhibitor, in patients with small-cell lung cancer and other solid tumors. J. Clin. Oncol. 29, 909–916. 10.1200/JCO.2010.31.6208 21282543 PMC4668282

[B28] Halgren T. A. Murphy R. B. Friesner R. A. Beard H. S. Frye L. L. Pollard W. T.. (2004). Glide: a new approach for rapid, accurate docking and scoring. 2. Enrichment factors in database screening. J. Med. Chem. 47, 1750–1759. 10.1021/jm030644s 15027866

[B29] Halim S. A. Khan S. Khan A. Wadood A. Mabood F. Hussain J.. (2017). Targeting dengue virus NS-3 helicase by ligand based pharmacophore modeling and structure based virtual screening. Front. Chem. 5:88. 10.3389/fchem.2017.00088 29164104 PMC5671650

[B30] Hanahan D. Weinberg R. A. (2000). The hallmarks of cancer. Cell 100, 57–70. 10.1016/S0092-8674(00)81683-9 10647931

[B31] Hanahan D. Weinberg R. A. (2011). Hallmarks of cancer: the next generation. Cell 144, 646–674. 10.1016/j.cell.2011.02.013 21376230

[B32] Hoover W. G. (1985). Canonical dynamics: equilibrium phase-space distributions. Phys. Rev. A 31:1695. 10.1103/PhysRevA.31.1695 9895674

[B33] Igney F. H. Krammer P. H. (2002). Death and anti-death: tumour resistance to apoptosis. Nat. Rev. Cancer 2, 277–288. 10.1038/nrc776 12001989

[B34] Is Y. S. Durdagi S. Aksoydan B. Yurtsever M. (2018). Proposing novel MAO-B hit inhibitors using multidimensional molecular modeling approaches and application of binary QSAR models for prediction of their therapeutic activity, pharmacokinetic and toxicity properties. ACS Chem. Neurosci. 9, 1768–1782. 10.1021/acschemneuro.8b00095 29671581

[B35] Jacobson M. P. Pincus D. L. Rapp C. S. Day T. J. Honig B. Shaw D. E.. (2004). A hierarchical approach to all-atom protein loop prediction. Proteins 55, 351–367. 10.1002/prot.10613 15048827

[B36] Kanan T. Kanan D. Erol I. Yazdi S. Stein M. Durdagi S. (2019). Targeting the NF-κB/IκBα complex via fragment-based E-Pharmacophore virtual screening and binary QSAR models. J. Mol. Graph. Modell. 86, 264–277. 10.1016/j.jmgm.2018.09.014 30415122

[B37] Kollek M. Müller A. Egle A. Erlacher M. (2016). Bcl-2 proteins in development, health, and disease of the hematopoietic system. FEBS J. 283, 2779–2810. 10.1111/febs.13683 26881825

[B38] Kumar Yadav D. Dhawan S. Chauhan A. Qidwai T. Sharma P. Singh Bhakuni R.. (2014a). QSAR and docking based semi-synthesis and *in vivo* evaluation of artemisinin derivatives for antimalarial activity. Curr. Drug Targets 15, 753–761. 10.2174/1389450115666140630102711 24975562

[B39] Kumar Yadav D. Kalani K. K. Singh A. Khan F. K. Srivastava S. Pant A. B. (2014b). Design, synthesis and *in vitro* evaluation of 18β-glycyrrhetinic acid derivatives for anticancer activity against human breast cancer cell line MCF-7. Curr. Med. Chem. 21, 1160–1170. 10.2174/09298673113206660330 24180274

[B40] Kumar Yadav D. Kalani K. Khan F. Kumar Srivastava S. (2013). QSAR and docking based semi-synthesis and *in vitro* evaluation of 18 β-glycyrrhetinic acid derivatives against human lung cancer cell line A-549. Med. Chem. 9, 1073–1084. 10.2174/1573406411309080009 23675978

[B41] Lessene G. Czabotar P. E. Colman P. M. (2008). BCL-2 family antagonists for cancer therapy. Nat. Rev. Drug Discov. 7, 989–1000. 10.1038/nrd2658 19043450

[B42] Li Y. Han L. Liu Z. Wang R. (2014). Comparative assessment of scoring functions on an updated benchmark: 2. Evaluation methods and general results. J. Chem. Inf. Model. 54, 1717–1736. 10.1021/ci500081m 24708446

[B43] Lionta E. Spyrou G. K. Vassilatis D. Cournia Z. (2014). Structure-based virtual screening for drug discovery: principles, applications and recent advances. Curr. Top. Med. Chem. 14, 1923–1938. 10.2174/1568026614666140929124445 25262799 PMC4443793

[B44] Martyna G. J. Tobias D. J. Klein M. L. (1994). Constant pressure molecular dynamics algorithms. J. Chem. Phys. 101, 4177–4189. 10.1063/1.467468

[B45] Mirza S. B. Lee R. C. H. Chu J. J. H. Salmas R. E. Mavromoustakos T. Durdagi S. (2018). Discovery of selective dengue virus inhibitors using combination of molecular fingerprint-based virtual screening protocols, structure-based pharmacophore model development, molecular dynamics simulations and *in vitro* studies. J. Mol. Graph. Modell. 79, 88–102. 10.1016/j.jmgm.2017.10.010 29156382

[B46] Mohamad Rosdi M. N. Mohd Arif S. Abu Bakar M. H. Razali S. A. Mohamed Zulkifli R. Ya'akob H. (2018). Molecular docking studies of bioactive compounds from Annona muricata Linn as potential inhibitors for Bcl-2, Bcl-w and Mcl-1 antiapoptotic proteins. Apoptosis 23, 27–40. 10.1007/s10495-017-1434-7 29204721

[B47] Mollica A. Zengin G. Durdagi S. Ekhteiari Salmas R. Macedonio G. Stefanucci A.. (2019). Combinatorial peptide library screening for discovery of diverse α-glucosidase inhibitors using molecular dynamics simulations and binary QSAR models. J. Biomol. Struct. Dyn. 37, 726–740. 10.1080/07391102.2018.1439403 29421954

[B48] Nosé S. (1984). A unified formulation of the constant temperature molecular dynamics methods. J. Chem. Phys. 81, 511–519. 10.1063/1.447334

[B49] Oltersdorf T. Elmore S. W. Shoemaker A. R. Armstrong R. C. Augeri D. J. Belli B. A.. (2005). An inhibitor of Bcl-2 family proteins induces regression of solid tumours. Nature 435, 677–681. 10.1038/nature03579 15902208

[B50] Petros A. M. Medek A. Nettesheim D. G. Kim D. H. Yoon H. S. Swift K.. (2001). Solution structure of the antiapoptotic protein bcl-2. Proc. Natl Acad. Sci. U.S.A. 98, 3012–3017. 10.1073/pnas.041619798 11248023 PMC30598

[B51] Rastelli G. Degliesposti G. Del Rio A. Sgobba M. (2009). Binding estimation after refinement, a new automated procedure for the refinement and rescoring of docked ligands in virtual screening. Chem. Biol. Drug Des. 73:283–86. 10.1111/j.1747-0285.2009.00780.x 19207463

[B52] Rastelli G. Pinzi L. (2019). Refinement and rescoring of virtual screening results. Front. Chem. 7:498. 10.3389/fchem.2019.00498 31355188 PMC6637856

[B53] Reed J. C. (2002). Apoptosis-based therapies. Nat. Rev. Drug Discov. 1, 111–121. 10.1038/nrd726 12120092

[B54] Rodrigues M. J. Slusarczyk S. Pecio Ł. Matkowski A. Salmas R. E. Durdagi S. (2018). *In vitro* and *in silico* approaches to appraise *Polygonum maritimum* L. as a source of innovative products with anti-ageing potential. Indust. Crops Products 111, 391–399. 10.1016/j.indcrop.2017.10.046

[B55] Salmas R. E. Seeman P. Aksoydan B. Erol I. Kantarcioglu I. Stein M.. (2017). Analysis of the glutamate agonist LY404,039 binding to nonstatic dopamine receptor D2 dimer structures and consensus docking. ACS Chem. Neurosci. 8, 1404–1415. 10.1021/acschemneuro.7b00070 28272861

[B56] Sastry G. M. Adzhigirey M. Day T. Annabhimoju R. Sherman W. (2013). Protein and ligand preparation: parameters, protocols, and influence on virtual screening enrichments. J. Comput. Aided Mol. Des. 27, 221–234. 10.1007/s10822-013-9644-8 23579614

[B57] Schrödinger Release 2015-2 (2015) LigPrep. New York, NY: Schrödinger, LLC.

[B58] Shan Y. Kim E. T. Eastwood M. P. Dror R. O. Seeliger M. A. Shaw D. E. (2011). How does a drug molecule find its target binding site? JACS 133, 9181–9183. 10.1021/ja202726y 21545110 PMC3221467

[B59] Shelley J. C. Cholleti A. Frye L. L. Greenwood J. R. Timlin M. R. Uchimaya M. (2007). Epik: a software program for pK a prediction and protonation state generation for drug-like molecules. J. Comput. Aided Mol. Des 21, 681–691. 10.1007/s10822-007-9133-z 17899391

[B60] Sherman W. Beard H. S. Farid R. (2006a). Use of an induced fit receptor structure in virtual screening. Chem. Biol. Drug Des. 67, 83–84. 10.1111/j.1747-0285.2005.00327.x 16492153

[B61] Sherman W. Day T. Jacobson M. P. Friesner R. A. Farid R. (2006b). Novel procedure for modeling ligand/receptor induced fit effects. J. Med. Chem. 49, 534–553. 10.1021/jm050540c 16420040

[B62] Soderquist R. S. Eastman A. (2016). BCL2 inhibitors as anticancer drugs: a plethora of misleading BH3 mimetics. Mol. Cancer Ther. 15, 2011–2017. 10.1158/1535-7163.MCT-16-0031 27535975 PMC5010924

[B63] Souers A. J. Leverson J. D. Boghaert E. R. Ackler S. L. Catron N. D. Chen J.. (2013). ABT-199, a potent and selective BCL-2 inhibitor, achieves antitumor activity while sparing platelets. Nat. Med 19, 202–208. 10.1038/nm.3048 23291630

[B64] Tse C. Shoemaker A. R. Adickes J. Anderson M. G. Chen J. Jin S.. (2008). ABT-263: a potent and orally bioavailable Bcl-2 family inhibitor. Cancer Res. 68, 3421–3428. 10.1158/0008-5472.CAN-07-5836 18451170

[B65] Verma S. Singh A. Mishra A. (2015). Complex disruption effect of natural polyphenols on Bcl-2-Bax: molecular dynamics simulation and essential dynamics study. J. Biomol. Struct. Dyn. 33, 1094–1106. 10.1080/07391102.2014.931823 24903407

[B66] Warren G. L. Andrews C. W. Capelli A.-M. Clarke B. LaLonde J. Lambert M. H.. (2006). A critical assessment of docking programs and scoring functions. J. Med. Chem. 49, 5912–5931. 10.1021/jm050362n 17004707

[B67] Wu H. Medeiros L. J. Young K. H. (2018). Apoptosis signaling and BCL-2 pathways provide opportunities for novel targeted therapeutic strategies in hematologic malignances. Blood Rev. 32, 8–28. 10.1016/j.blre.2017.08.004 28802908

[B68] Yang W. Soares J. Greninger P. Edelman E. J. Lightfoot H. Forbes S.. (2012). Genomics of Drug Sensitivity in Cancer (GDSC): a resource for therapeutic biomarker discovery in cancer cells. Nucleic Acids Res. 41, D955–D961. 10.1093/nar/gks1111 23180760 PMC3531057

[B69] Yoshino R. Yasuo N. Hagiwara Y. Ishida T. Inaoka D. K. Amano Y.. (2017). *In silico, in vitro*, X-ray crystallography, and integrated strategies for discovering spermidine synthase inhibitors for Chagas disease. Sci. Rep. 7:6666. 10.1038/s41598-017-06411-9 28751689 PMC5532286

[B70] Yoshino R. Yasuo N. Inaoka D. K. Hagiwara Y. Ohno K. Orita M.. (2015). Pharmacophore modeling for anti-chagas drug design using the fragment molecular orbital method. PLoS ONE 10:e0125829. 10.1371/journal.pone.0125829 25961853 PMC4427443

[B71] Youle R. J. Strasser A. (2008). The BCL-2 protein family: opposing activities that mediate cell death. Nat. Rev. Mol. Cell Biol. 9, 47–59. 10.1038/nrm2308 18097445

[B72] Zaka M. Abbasi B. H. Durdagi S. (2018). Proposing novel TNFα direct inhibitor Scaffolds using fragment-docking based e-pharmacophore modeling and binary QSAR-based virtual screening protocols pipeline. J. Mol. Graph. Modell. 85, 111–121. 10.1016/j.jmgm.2018.07.007 30149308

[B73] Zaka M. Abbasi B. H. Durdagi S. (2019). Novel tumor necrosis factor-α (TNF-α) inhibitors from small molecule library screening for their therapeutic activity profiles against rheumatoid arthritis using target-driven approaches and binary QSAR models. J. Biomol. Struct. Dyn. 37, 2464–2476. 10.1080/07391102.2018.1491423 30047845

[B74] Zhang H. Nimmer P. Tahir S. Chen J. Fryer R. Hahn K.. (2007). Bcl-2 family proteins are essential for platelet survival. Cell Death Differ. 14, 943–951. 10.1038/sj.cdd.4402081 17205078

