# Supplementary material for: Integrating Ligand and Target-Driven Based Virtual Screening Approaches With in vitro Human Cell Line Models and Time-Resolved Fluorescence Resonance Energy Transfer Assay to Identify Novel Hit Compounds Against BCL-2
Source: Front Chem. 2020 Apr 9;8:167. doi: 10.3389/fchem.2020.00167 (PMC7160371; doi:10.3389/fchem.2020.00167)
Supplement: Supplementary file 2 [file Data_Sheet_2.docx]

Supplementary Material


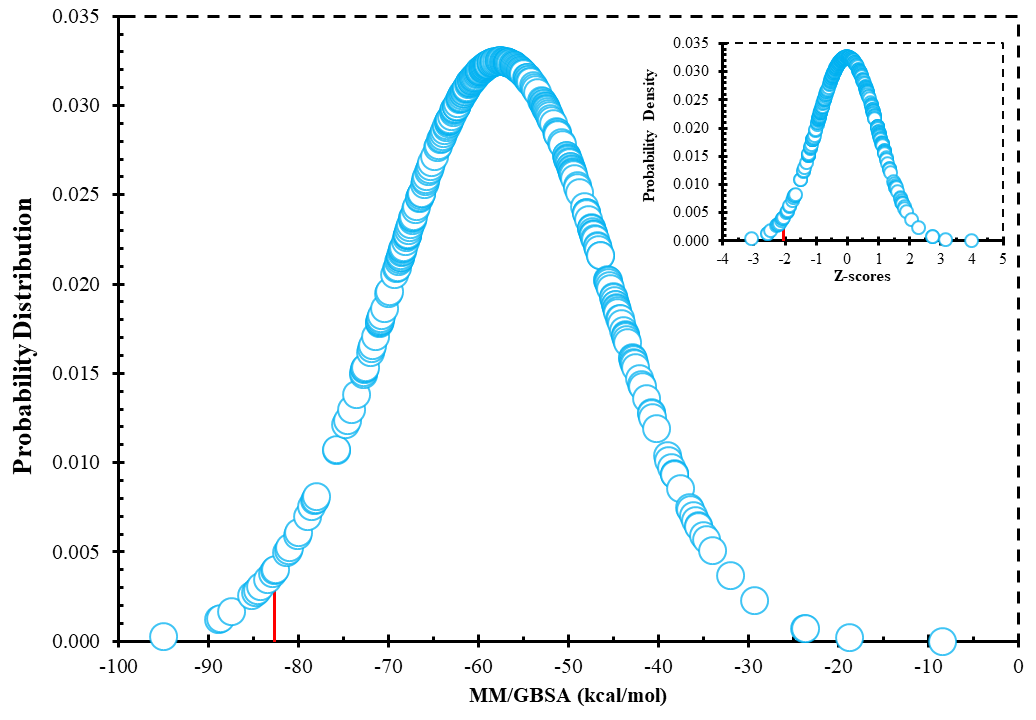
**Figure S1.** The normal distribution curves for MM/GBSA energies of 342 molecules selected after toxicity QSAR test.

**
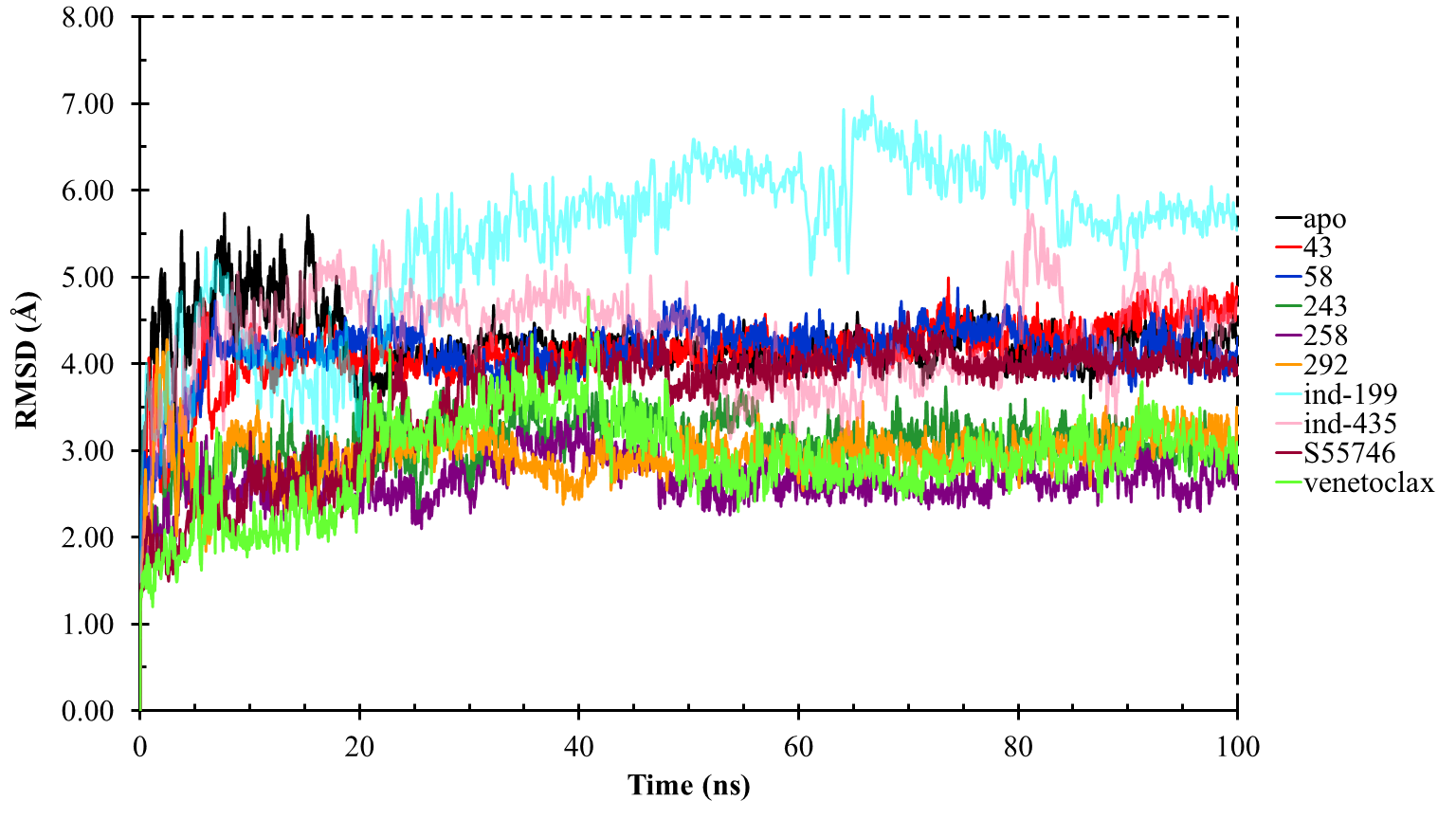
**


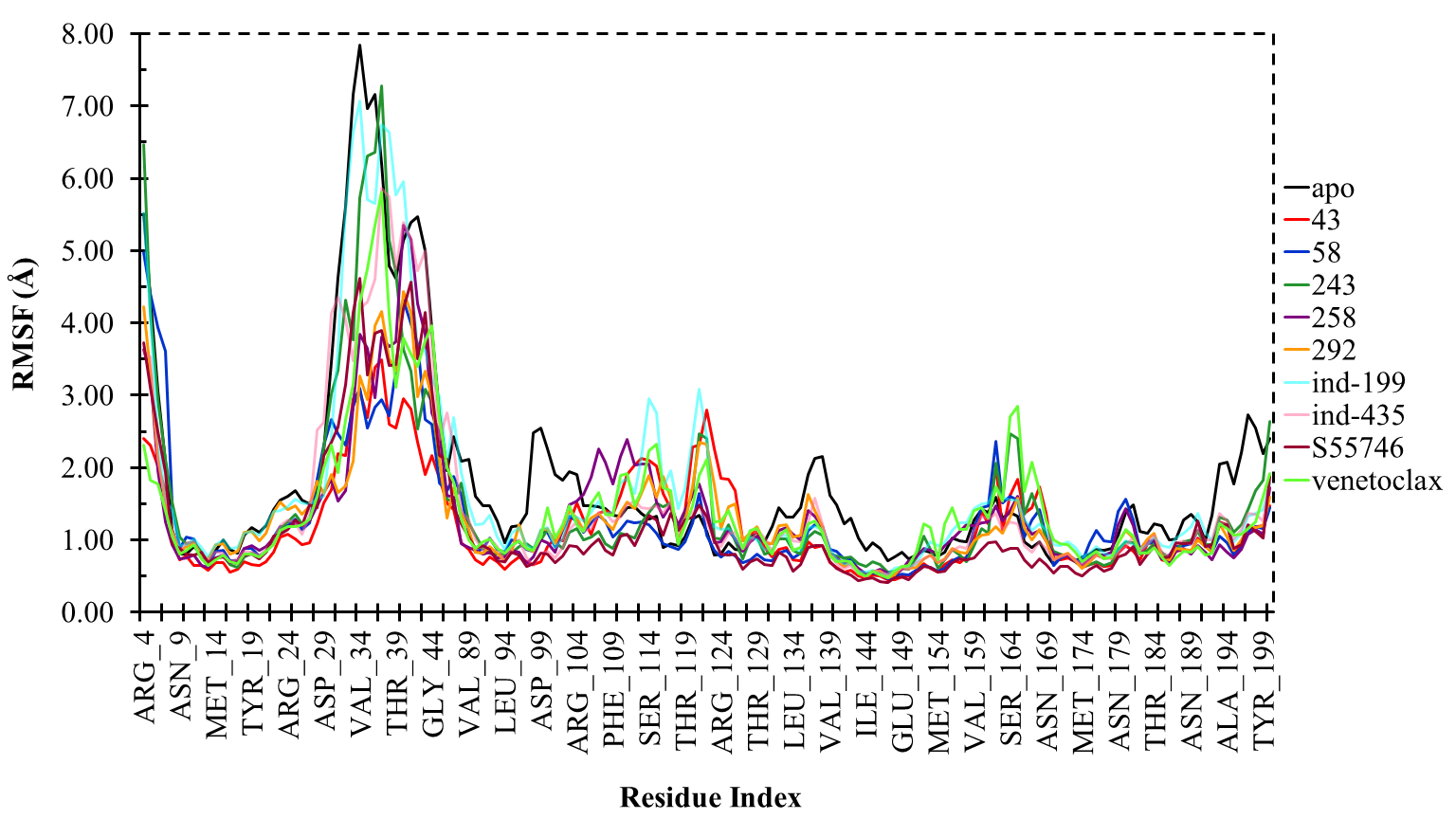
**Figure S2.** Protein RMSD graph of the Cα atoms of the BCL-2 obtained from 100 ns MD simulations.

**Figure S3.** Protein RMSF graph of the Cα atoms of the BCL-2 obtained from 100 ns MD simulations.


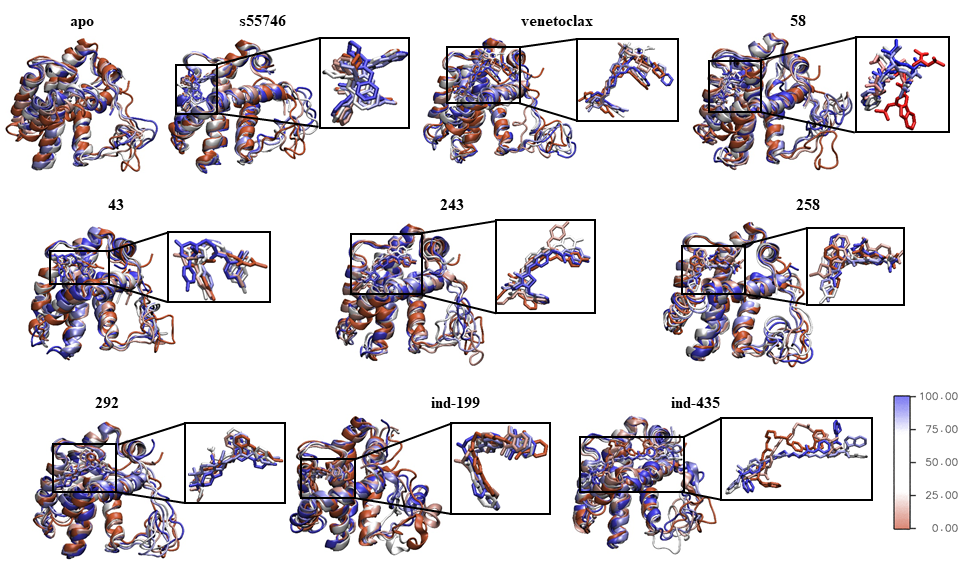
**Figure S4.** Conformational changes of the apo-state of the enzyme and hit ligands as well as reference molecules (venetoclax and S55746) bound states of the enzyme throughout the MD simulations.


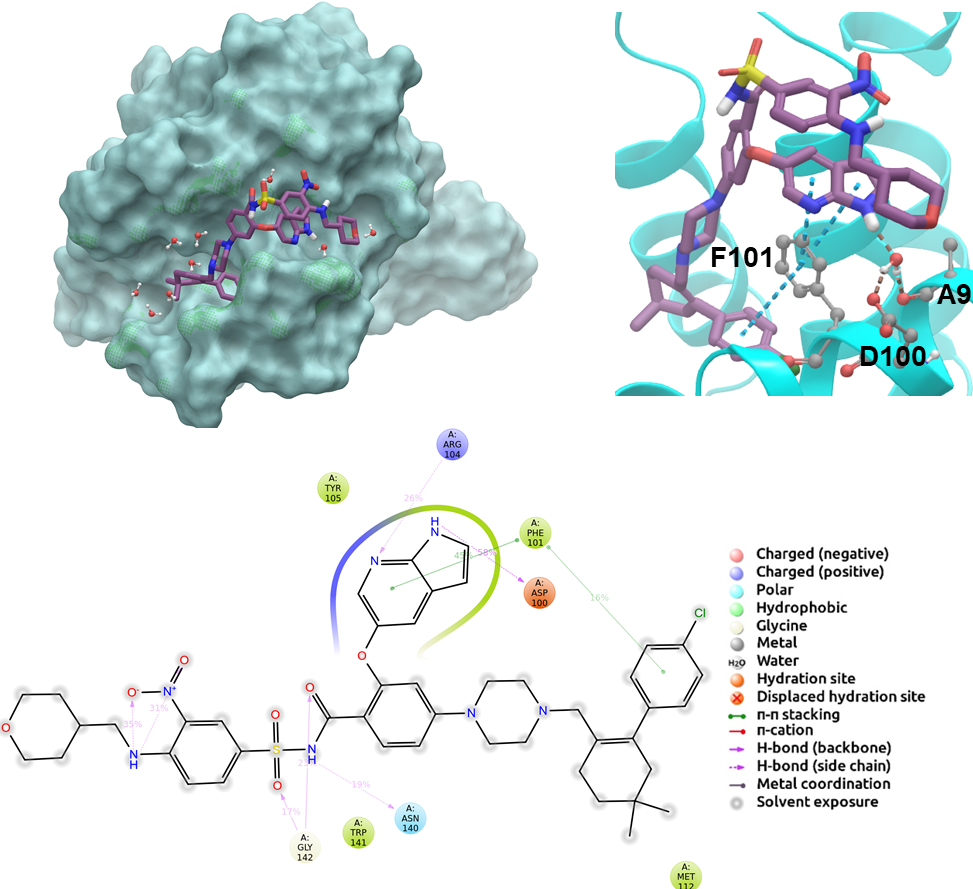


**Figure S5.** 2D and 3D ligand interactions diagrams of selected positive control molecule venetoclax at the binding pocket of BCL-2. Surface and ribbon representation are displayed for representative structure obtained from MD simulations while 2D interaction diagram shows the systematic details of protein-ligand interactions observed during MD.

**
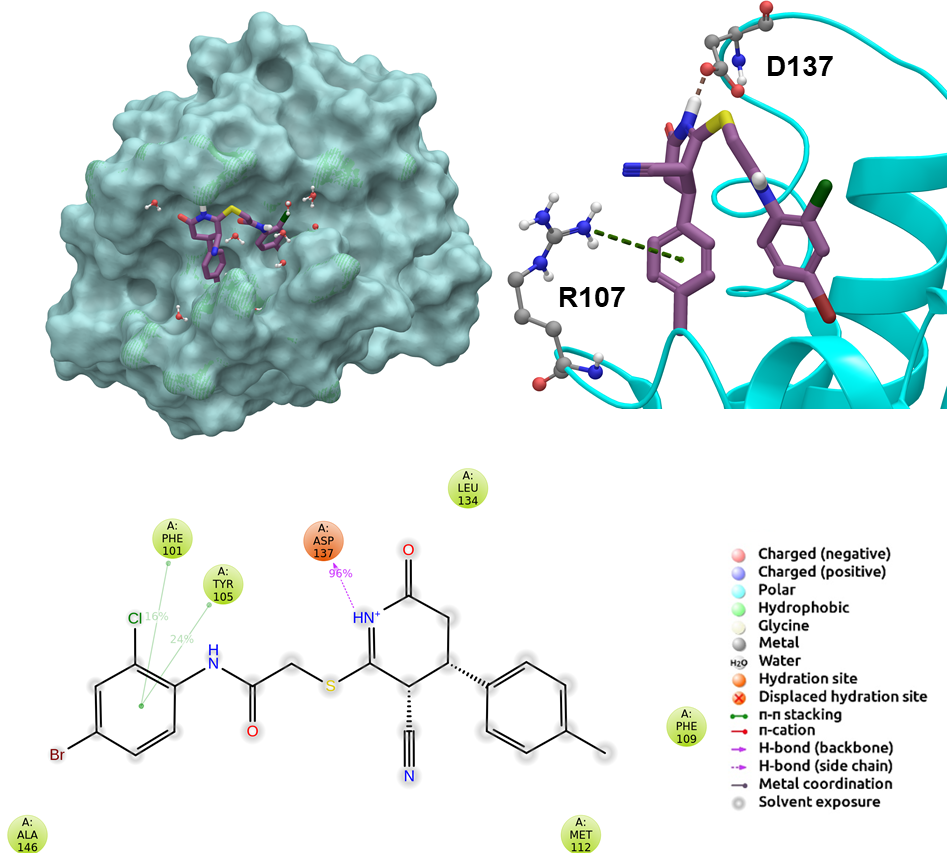
Figure S6**. 2D and 3D ligand interactions diagrams of selected molecule **43** at the binding pocket of BCL-2. Surface and ribbon representation are displayed for representative structure obtained from MD simulations while 2D interaction diagram shows the systematic details of protein-ligand interactions observed during MD.


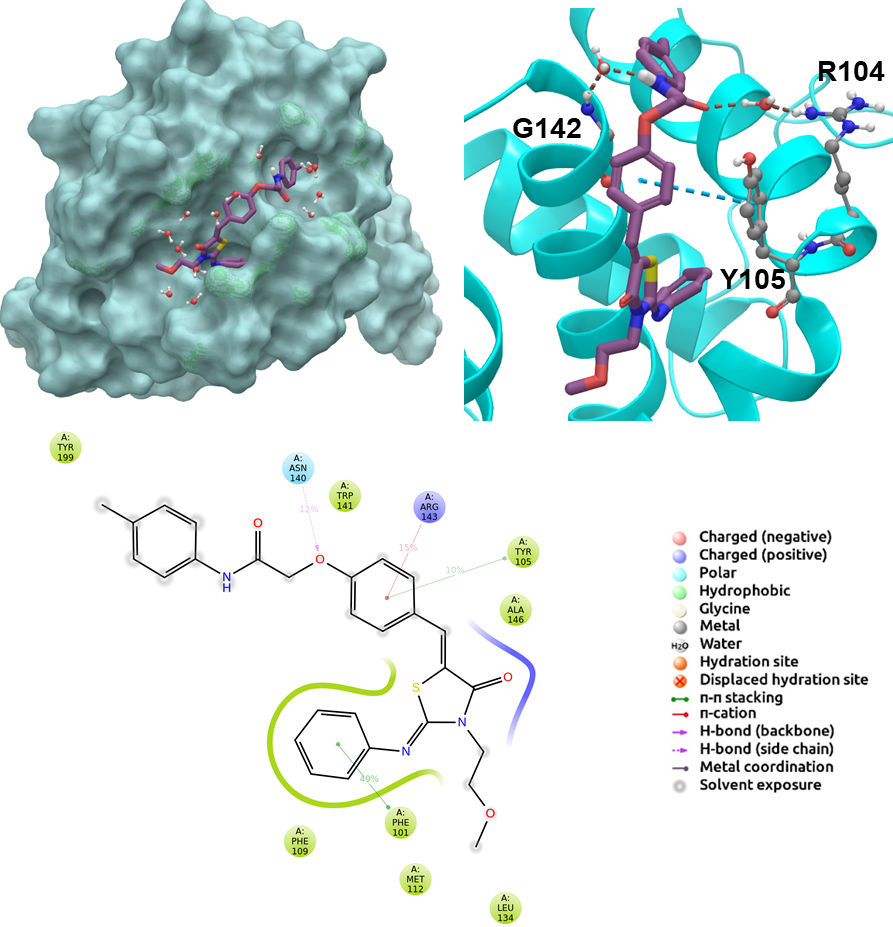
**Figure S7.** 2D and 3D ligand interactions diagrams of selected molecule 2**43** at the binding pocket of BCL-2. Surface and ribbon representation are displayed for representative structure obtained from MD simulations while 2D interaction diagram shows the systematic details of protein-ligand interactions observed during MD.


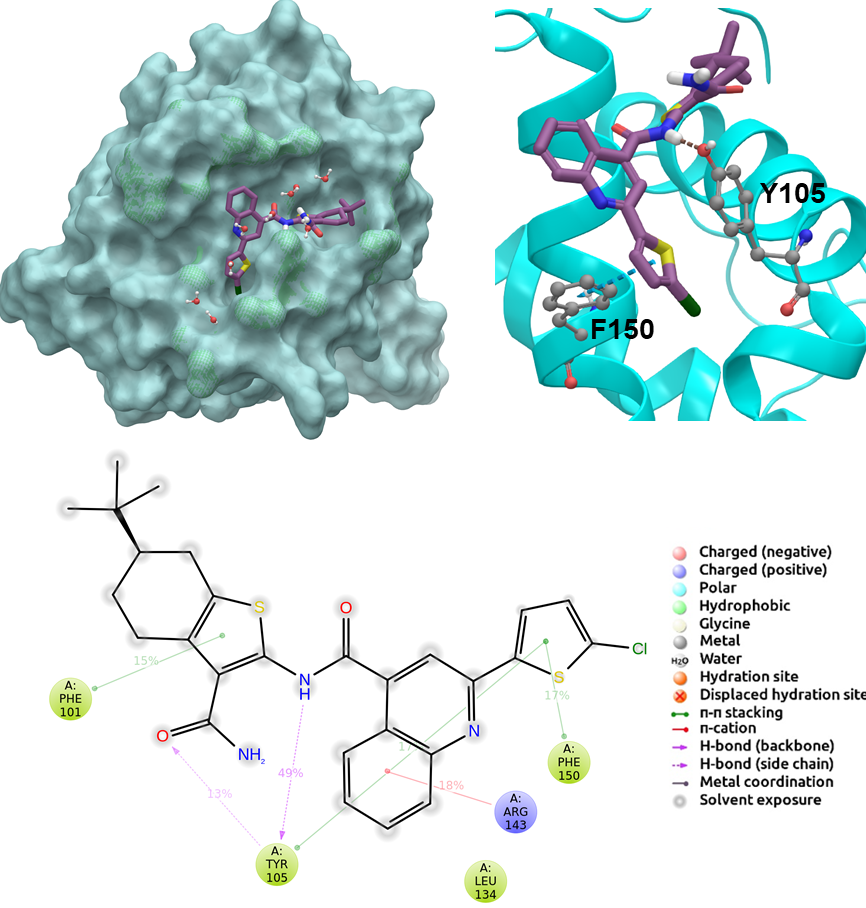
**Figure S8.** 2D and 3D ligand interactions diagrams of selected molecule **258** at the binding pocket of BCL-2. Surface and ribbon representation are displayed for representative structure obtained from MD simulations while 2D interaction diagram shows the systematic details of protein-ligand interactions observed during MD.


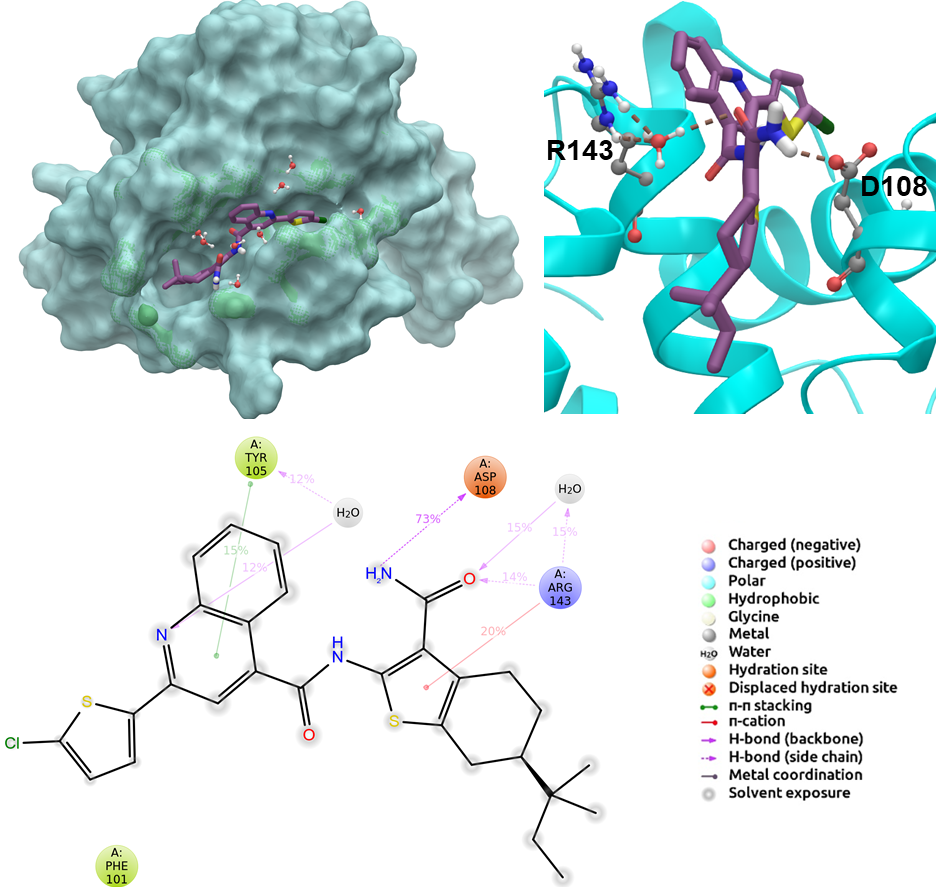


**Figure S9.** 2D and 3D ligand interactions diagrams of selected molecule **292** at the binding pocket of BCL-2. Surface and ribbon representation are displayed for representative structure obtained from MD simulations while 2D interaction diagram shows the systematic details of protein-ligand interactions observed during MD.


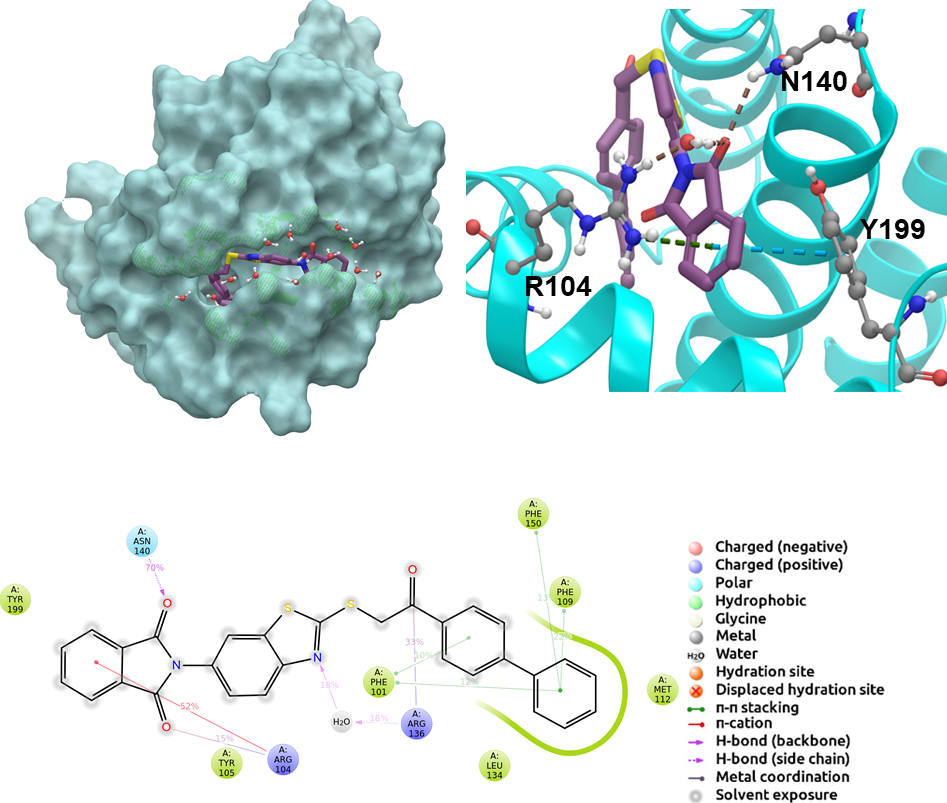
**Figure S10.** 2D and 3D ligand interactions diagrams of selected molecule **ind-199** at the binding pocket of BCL-2. Surface and ribbon representation are displayed for representative structure obtained from MD simulations while 2D interaction diagram shows the systematic details of protein-ligand interactions observed during MD.


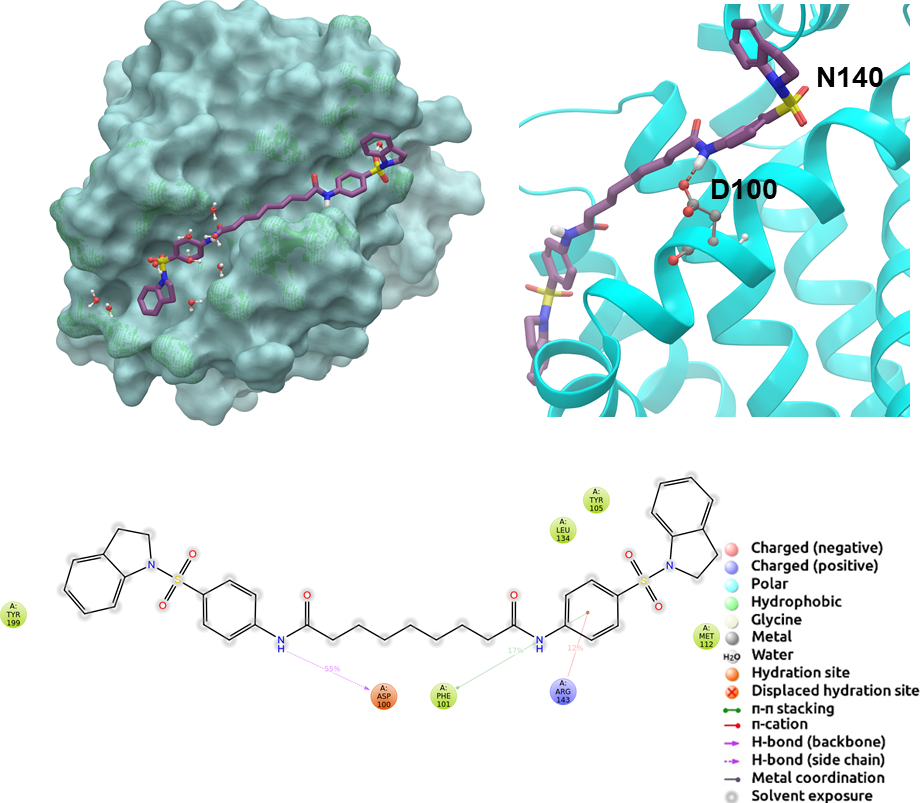


**Figure S11.** 2D and 3D ligand interactions diagrams of selected molecule **ind-435** at the binding pocket of BCL-2. Surface and ribbon representation are displayed for representative structure obtained from MD simulations while 2D interaction diagram shows the systematic details of protein-ligand interactions observed during MD

**
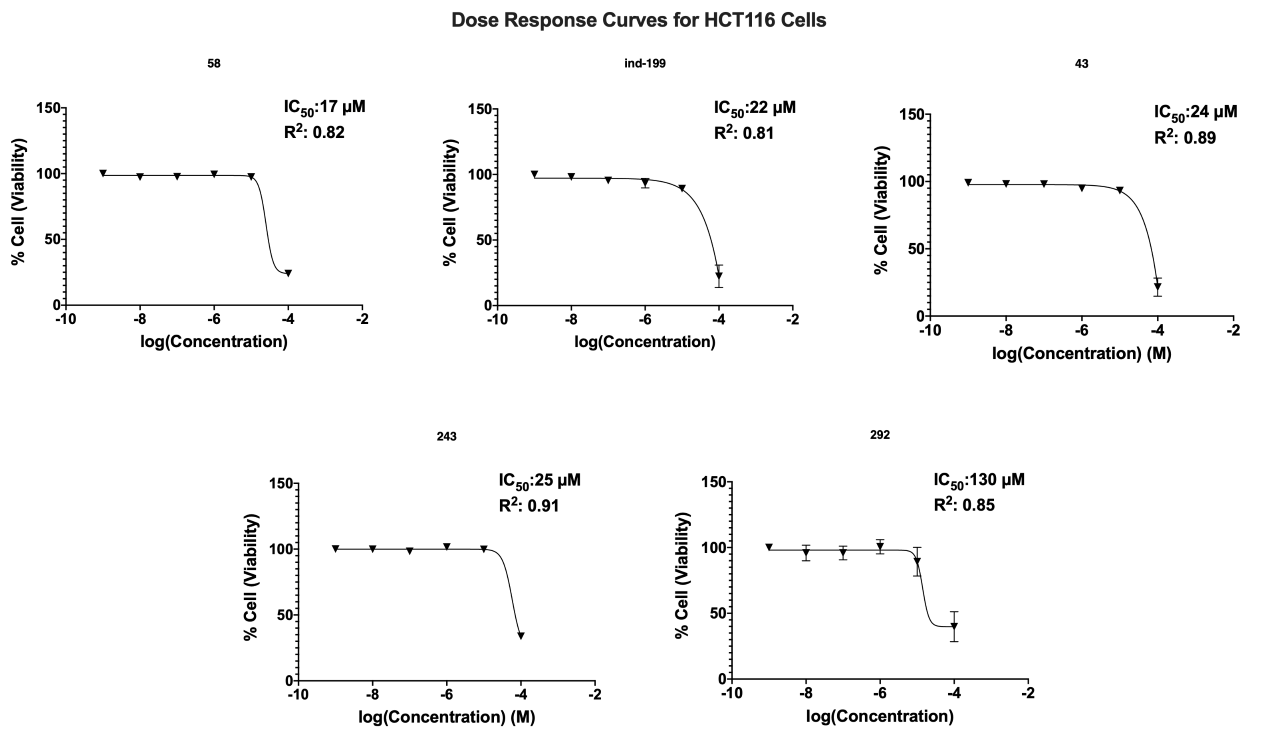
Figure S12**. Dose-response curves and calculated IC_50_ and R^2^ values on cell viability of HCT116 cells treated with active molecules.


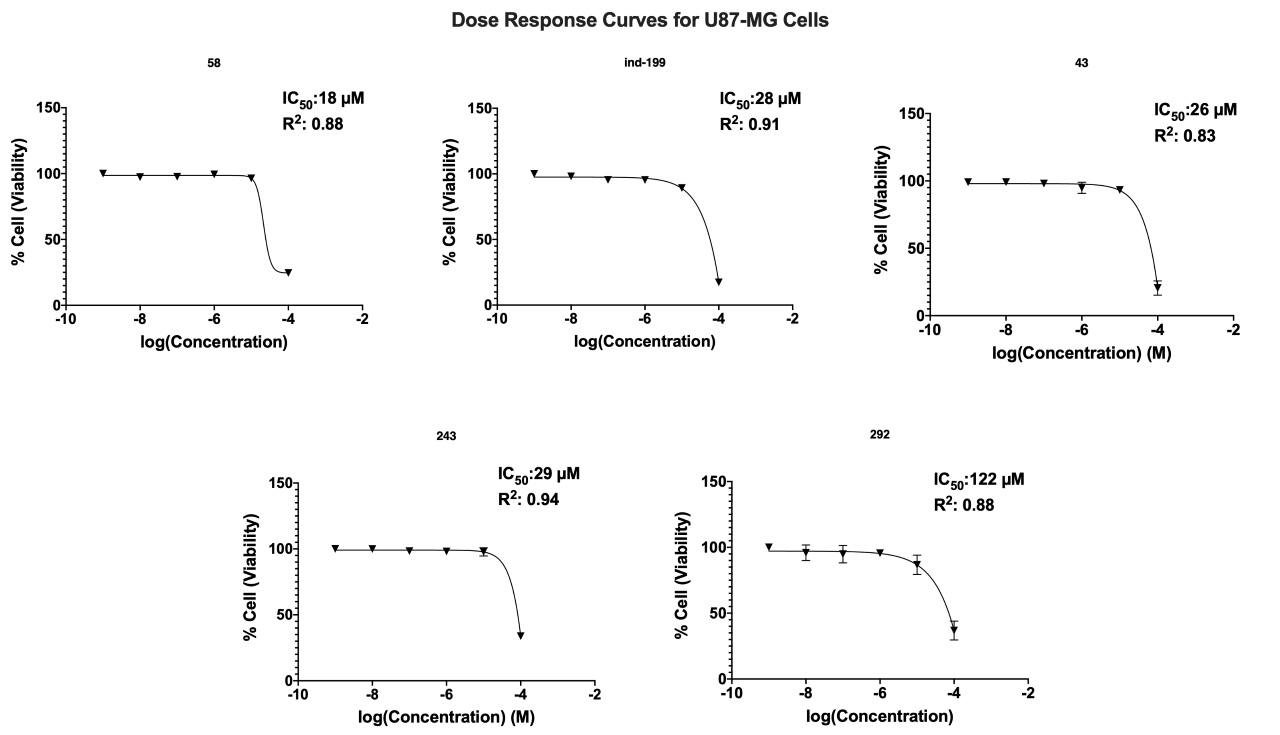


**Figure S13**. Dose-response curves and calculated IC_50_ and R^2^ values on cell viability of U87-MG cells treated with active molecules.


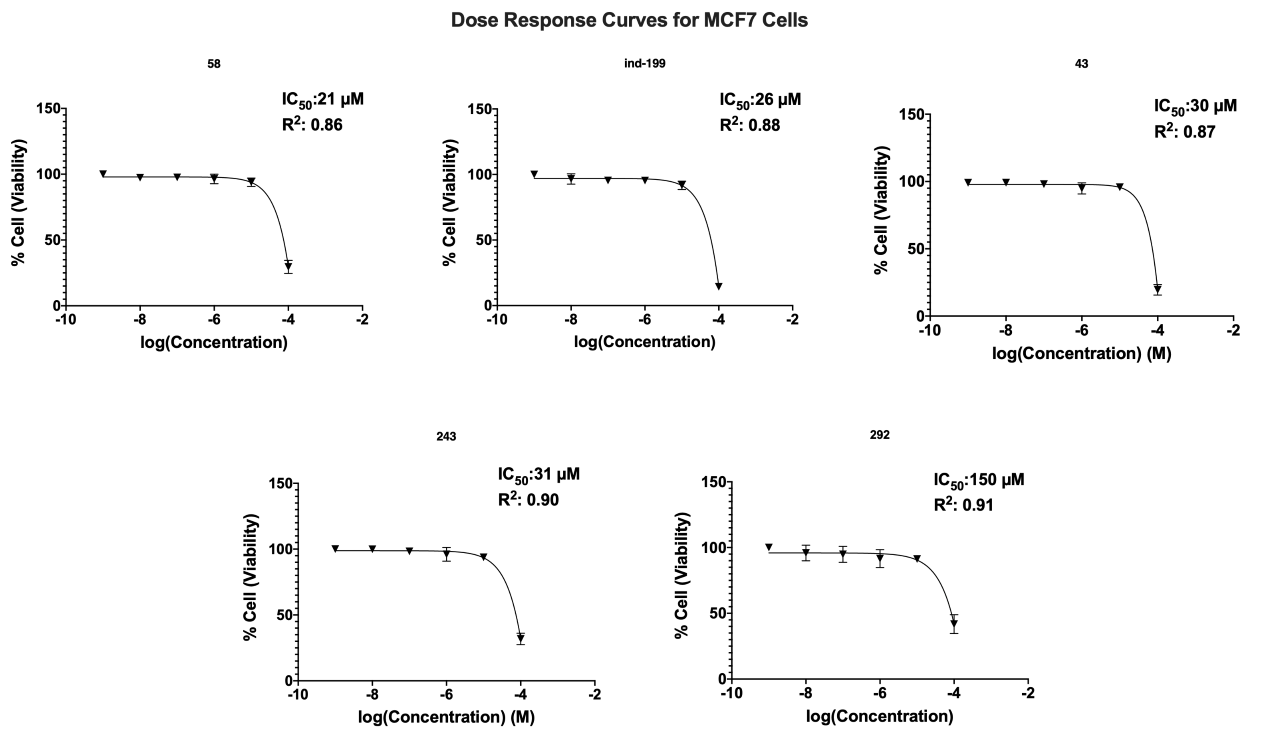


**Figure S14**. Dose-response curves and calculated IC_50_ and R^2^ values on cell viability of MCF7 cells treated with active molecules.


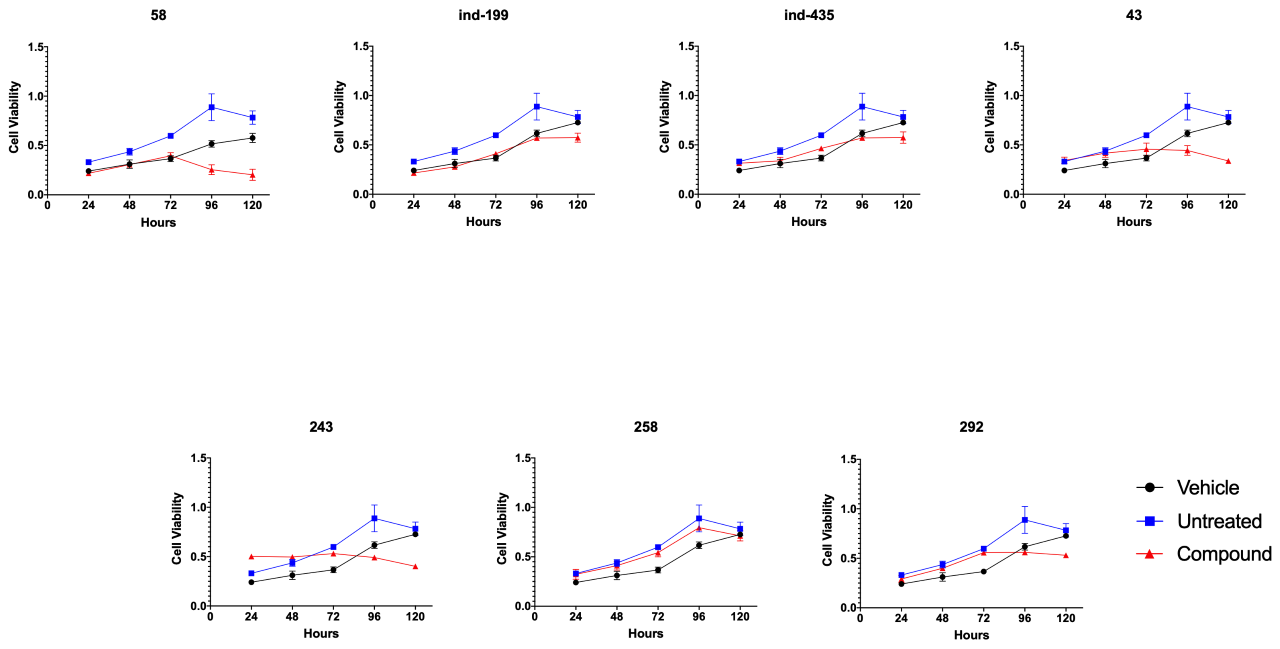


**Figure S15.** MTT cell proliferation assay was performed for U87-MG cells. Tested concentration for each drug was 100 μM. Vehicle group was treated with %2 DMSO, and untreated represents no molecule treated group. Molecule responses evaluated by cell viability which is obtained by spectrophotometric analysis of cells upon MTT treatment at day 24h ,48h, 72h, 96h and 120h.


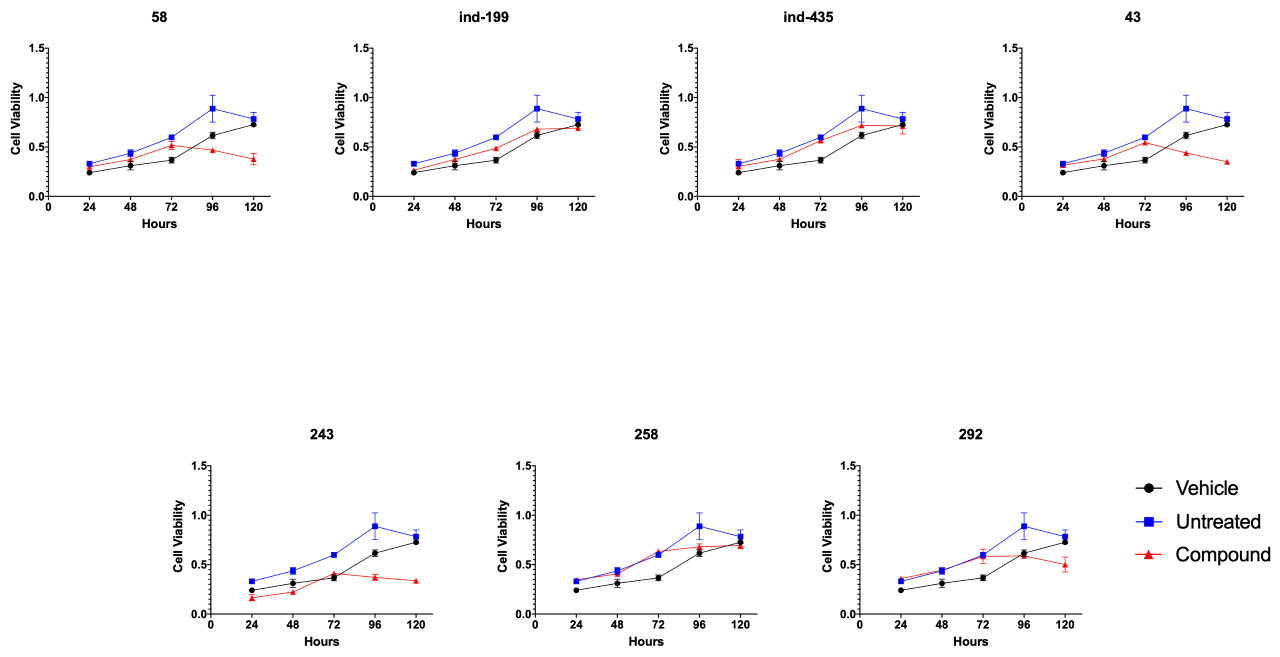


**Figure S16.** MTT cell proliferation assay was performed for MCF7 cells. Tested concentration for each drug was 100 μM. Vehicle group was treated with %2 DMSO, and untreated represents no molecule treated group. Molecule responses evaluated by cell viability which is obtained by spectrophotometric analysis of cells upon MTT treatment at day 24h ,48h, 72h, 96h and 120h.


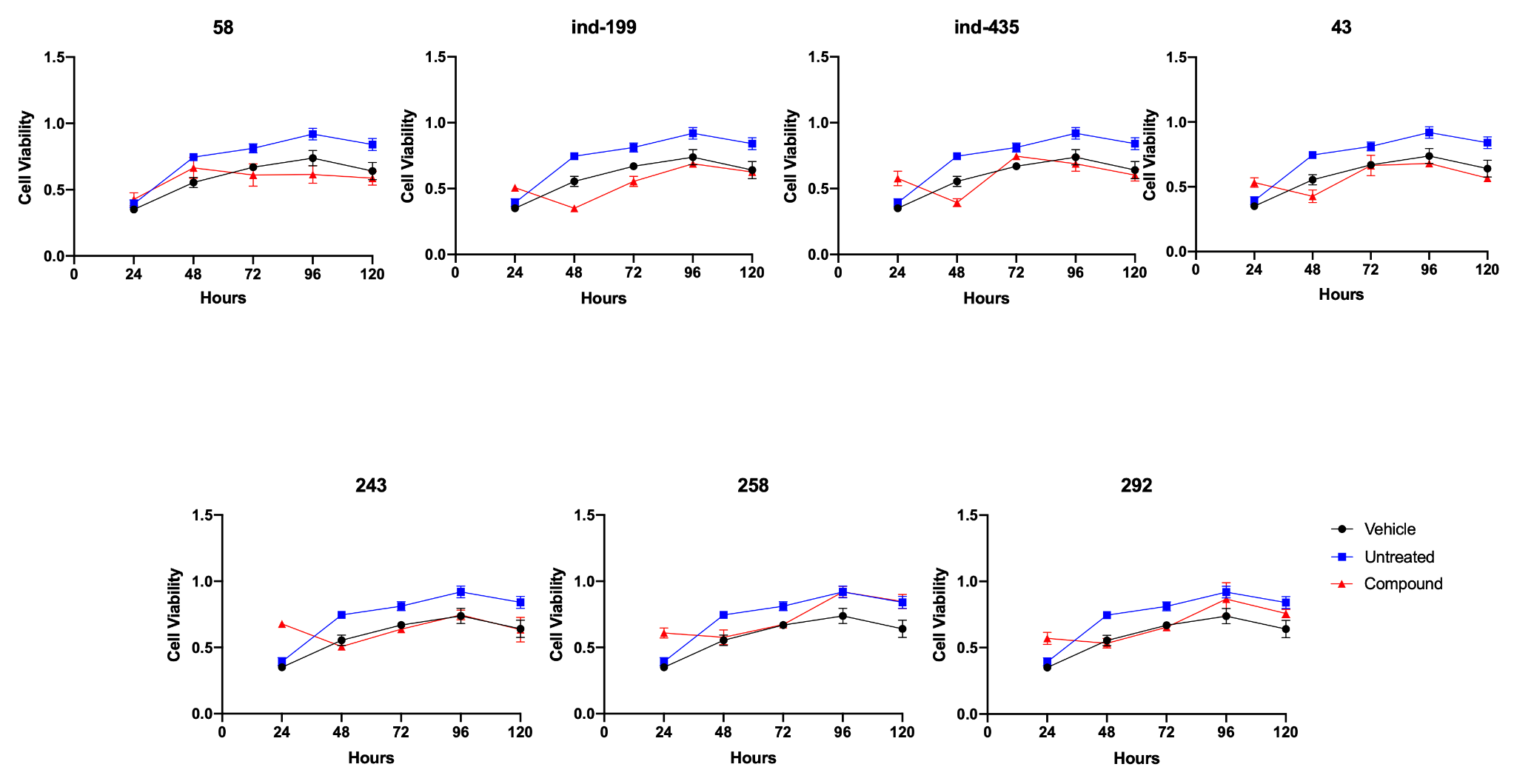


**Figure S17.** MTT cell proliferation assay was performed for HUVEC cells as noncancer control group. Tested concentration for each drug was 100 μM. Vehicle group was treated with %2 DMSO, and untreated represents no molecule treated group. Molecule responses evaluated by cell viability which is obtained by spectrophotometric analysis of cells upon MTT treatment at day 24h ,48h, 72h, 96h and 120h. None of the molecules showed significant difference in terms of cell viability on HUVEC cells. Statistical significance in graphs was determined by comparing each treatment group with DMSO control using ANOVA testing and significance is considered as p<0.001. Error bars show standard deviation.
