# Supplementary material for: Integrating Ligand and Target-Driven Based Virtual Screening Approaches With in vitro Human Cell Line Models and Time-Resolved Fluorescence Resonance Energy Transfer Assay to Identify Novel Hit Compounds Against BCL-2
Source: Front Chem. 2020 Apr 9;8:167. doi: 10.3389/fchem.2020.00167 (PMC7160371; doi:10.3389/fchem.2020.00167)
Supplement: Supplementary file 3 [file Data_Sheet_3.docx]

| **Table S1.** The Specs ID, 2D structures, docking scores and ligand efficiency for compounds selected based on docking approach with reference molecules. (Bold ones are selected for *in vitro* analysis). | | |
| --- | --- | --- |
| Molecule No. (Specs ID) | 2D Structure | Docking Score / Ligand efficiency * |
| **243 (AN-698/40780701)** | 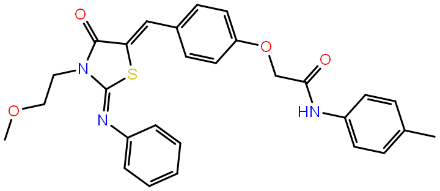 | **-13.21 / -0.37** |
| **58 (AJ-292/12931005)** | 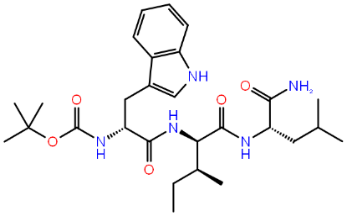 | **-12.82 / -0.34** |
| **43 (AO-081/41887762)** | 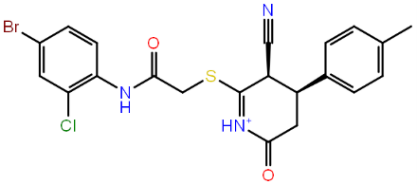 | **-12.56 / -0.43** |
| 55 (AO-081/41887745) | 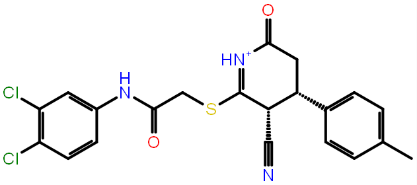 | -12.52 / -0.43 |
| 142 (AE-848/41827527) | 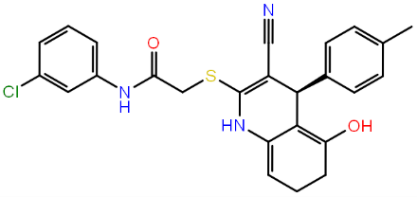 | -12.46 / -0.39 |
| Venetoclax | 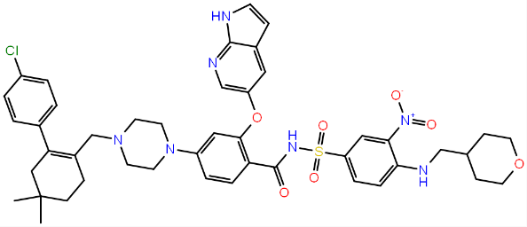 | -15.46 / -0.24 |
| S55746 (CHEMBL3958369)^†^ | 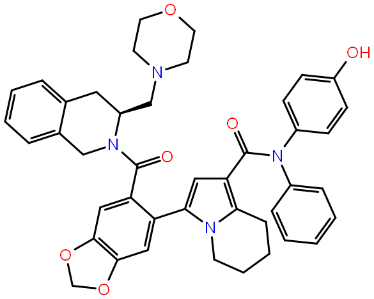 | -13.65^†^ / -0.26 |
| * Values in kcal/mol  ^†^The docking score for this reference molecule is calculated by using “score only” option of Glide/SP. | | |

**Table S2.** The Specs ID, 2D structure and MM/GBSA average values of compounds selected after short MD simulations based on their average MM/GBSA calculations. (Bold ones are selected for *in vitro* analysis).

| Compounds (Specs ID) | 2D Structure | MM/GBSA (kcal/mol) |
| --- | --- | --- |
| 315 (AH-487/40935580) | 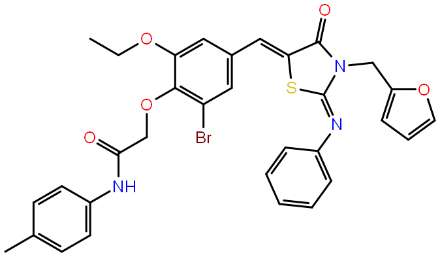 | -107.62 ± 6.60 |
| **243 (AN-698/40780701)** | 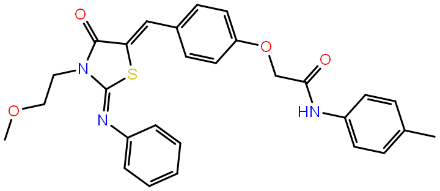 | **-102.65 ± 4.56** |
| 222 (AF-399/15128576) | 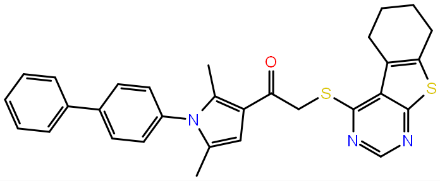 | -89.26 ± 3.13 |
| **292 (AK-968/11842328)** | **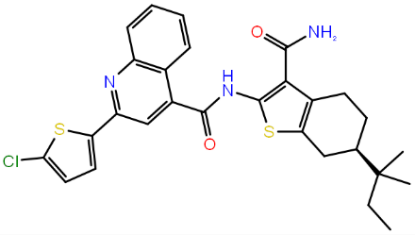** | **-88.93 ± 5.46** |
| 329 (AK-968/12096309) | 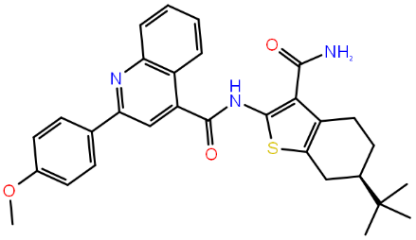 | -85.16 ± 6.19 |
| **258 (AK-968/12163470)** | **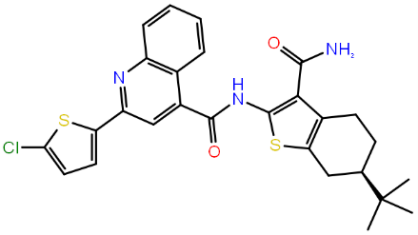** | **-83.50 ± 5.46** |
| 147 (AF-399/40920937) | 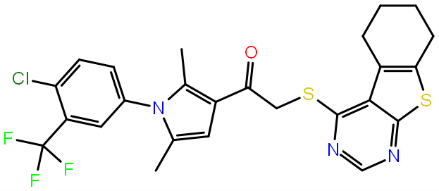 | -74.19 ± 4.32 |
| 63 (AG-690/40750327) | 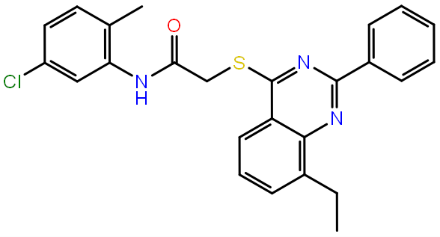 | -72.42 ± 6.73 |
| 70 (AG-690/40775494) | 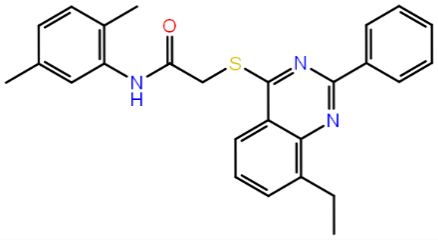 | -72.29 ± 4.06 |
| 146 (AK-968/12117056) | 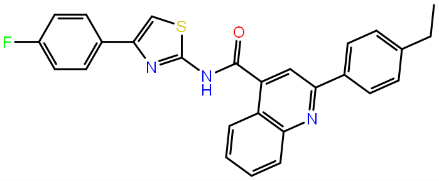 | -71.13 ± 5.76 |
| 342 (AK-968/41017038) | 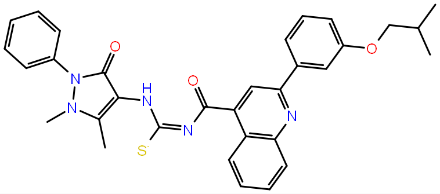 | -59.65 ± 4.52 |
| 225 (AK-968/15604755) | 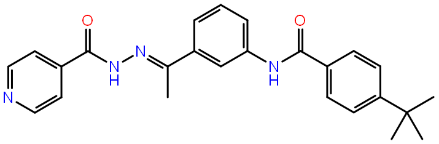 | -57.58 ± 9.88 |
| Venetoclax | 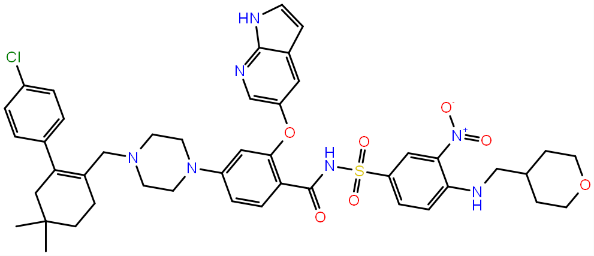 | -116.31 ± 9.20 |
| S55746 (CHEMBL3958369) | 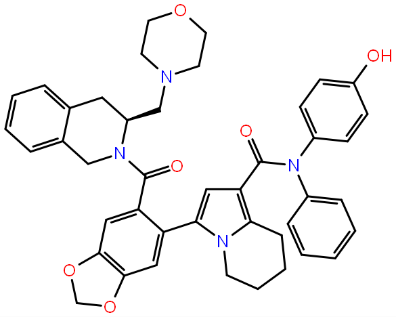 | -101.43 ± 4.20 |

**Table S3.** The Specs ID, 2D structure and MM/GBSA average values of selected indol phase containing compounds with reference molecules. (Bold ones are selected for *in vitro* analysis).

| Compounds (Specs ID) | 2D Structure | MM/GBSA (kcal/mol) |
| --- | --- | --- |
| **ind-199 (AG-205/12549135)** | **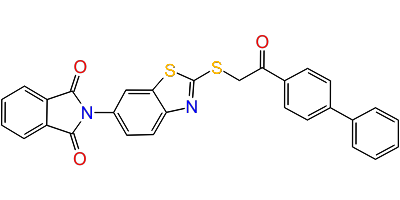** | **-107.41 ± 7.49** |
| ind-471 (AG-690/33095016) | **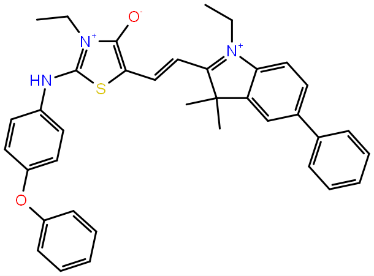** | -103.50 ± 5.35 |
| ind-617 (AF-399/15127076) | **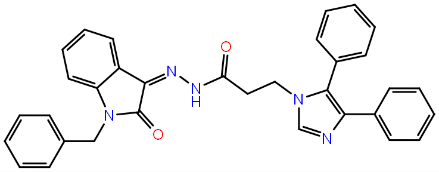** | -102.13 ± 4.80 |
| ind-1300 (AQ-088/42013632) | **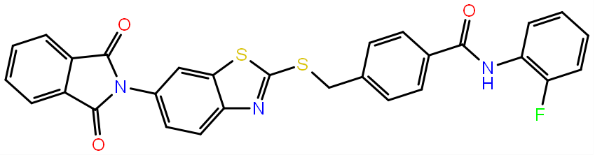** | -94.61 ± 7.45 |
| **ind-435 (AN-329/13484046)** | **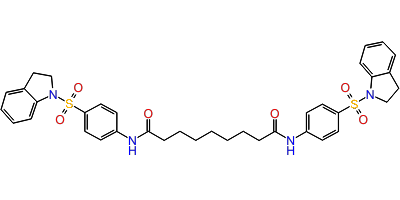** | **-94.17 ± 7.28** |
| ind-1286 (AQ-088/42013629) | **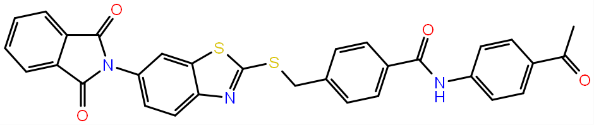** | -92.64 ± 4.70 |
| ind-717 (AM-900/15548155) | **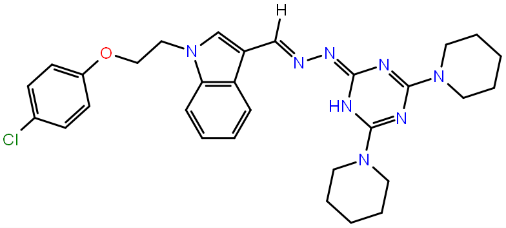** | -90.35 ± 8.53 |
| ind-1179 (AG-690/36164031) | **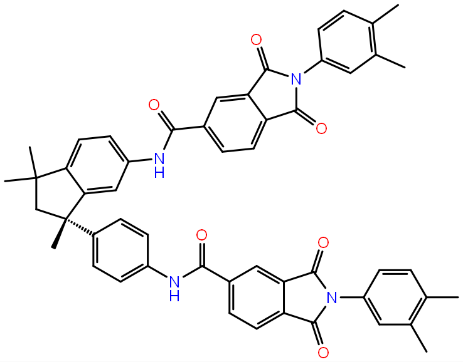** | -89.52 ± 13.67 |
| ind-687 (AO-299/15047132) | **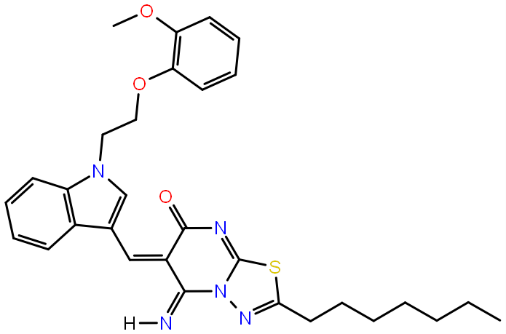** | -88.26 ± 7.77 |
| ind-645 (AO-299/15047107) | **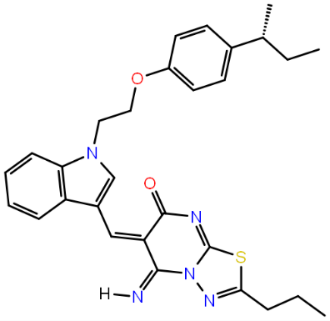** | -87.50 ± 6.16 |
| Venetoclax | **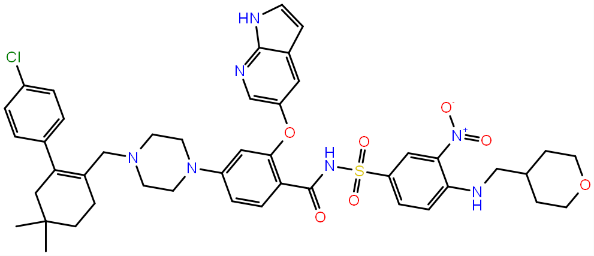** | -116.31 ± 9.20 |
| S55746 (CHEMBL3958369) | **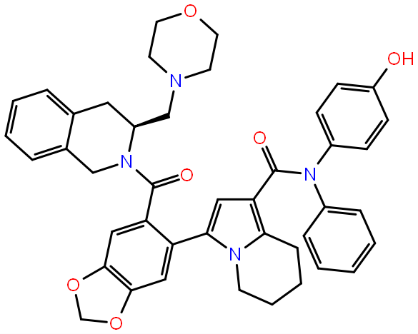** | -101.43 ± 4.20 |
